# A Novel and Efficient Platform for Discovering Noncanonical Quorum-Quenching Proteins

**DOI:** 10.1128/spectrum.03437-22

**Published:** 2022-12-07

**Authors:** Jinxing Liao, Zihan Li, Dan Xiong, Danyu Shen, Lu Wang, Xiaolong Shao, Tao Li, Guoliang Qian

**Affiliations:** a College of Plant Protection, Laboratory of Plant Immunity, Key Laboratory of Integrated Management of Crop Diseases and Pests, Nanjing Agricultural University, Nanjing, Jiangsu, People’s Republic of China; b Medical College, China Three Gorges University, Yichang, People’s Republic of China; c Shanghai Veterinary Research Institute, Chinese Academy of Agricultural Sciences, Shanghai, People’s Republic of China; Weizmann Institute of Science

**Keywords:** quorum sensing, quorum quenching, AHL synthase, AHL, *Lysobacter*, noncanonical quorum-quenching protein

## Abstract

Quorum sensing (QS) is a well-known chemical signaling system responsible for intercellular communication that is widespread in bacteria. Acyl-homoserine lactone (AHL) is the most-studied QS signal. Previously, bacterially encoded AHL-degrading enzymes were considered to be canonical quorum-quenching proteins that have been widely used to control pathogenic infections. Here, we report a novel platform that enabled the efficient discovery of noncanonical AHL quorum-quenching proteins. This platform initially asked bacteriologists to carry out comparative genomic analyses between phylogenetically related AHL-producing and non-AHL-producing members to identify genes that are conservatively shared by non-AHL-producing members but absent in AHL-producing species. These candidate genes were then introduced into recombinant AHL-producing E. coli to screen for target proteins with the ability to block AHL production. Via this platform, we found that non-AHL-producing *Lysobacter* containing numerous environmentally ubiquitous members encoded a conserved glycosyltransferase-like protein Le4759, which was experimentally shown to be a noncanonical AHL-quenching protein. Le4759 could not directly degrade exogenous AHL but rather recognized and altered the activities of multiple AHL synthases through protein-protein interactions. This versatile capability enabled Le4759 to block specific AHL synthase such as CarI from Pectobacterium carotovorum to reduce its protein abundance to suppress AHL synthesis, thereby impairing bacterial infection. Thus, this study provided bacteriologists with a unique platform to discover noncanonical quorum-quenching proteins that could be developed as promising next-generation drug candidates to overcome emerging bacterial antibiotic resistance.

**IMPORTANCE** Targeting and blocking bacterial quorum sensing (QS), the process known as quorum quenching (QQ) is an effective mean to control bacterial infection and overcome the emerging antibiotic resistance. Previously, diverse QS signal-degradation enzymes are identified as canonical QQ proteins. Here, we provided a novel and universal platform that enabled to discover previously unidentified noncanonical QQ proteins that were unable to degrade acyl-homoserine lactone (AHL) but could block AHL generation by recognizing multiple AHL synthases via direct protein-protein interactions. Our findings are believed to trigger broad interest for bacteriologists to identify potentially widely distributed noncanonical QQ proteins that have great potential for developing next-generation anti-infectious drugs.

## INTRODUCTION

Unlike eukaryotic organisms, bacteria are single-cell organisms. Therefore, it is interesting to understand how bacterial cells establish intraspecific or interspecific communication. Numerous earlier studies have shown that bacteria evolutionarily acquire a strategy called quorum sensing (QS) to communicate within or between bacterial species (1–4). Specifically, bacterial QS is associated with extracellular chemical signals (auto-inducers) produced by the bacteria themselves that can accumulate in the local environment to the levels required to regulate transcription of a specific gene (5–8). In general, Gram-positive bacteria use processed oligopeptides as autoinducer peptides (AIP) (9, 10), whereas some Gram-negative bacteria produce and utilize acylated homoserine lactones (AHLs) as QS signals that carry a core N-acylated homoserine-lactone ring and a flexible 4 to 18 carbon acyl chain (11–14). Many bacterial species use LuxI-type synthases, known as AHL synthase (15–17), to make these AHLs. Presence of threshold concentration of AHL and its binding to LuxR-type transcription factors result in modulation of multiple gene expression, cellular processes, and physiological activities regulated by AHL (18, 19).

**FIG 1:**
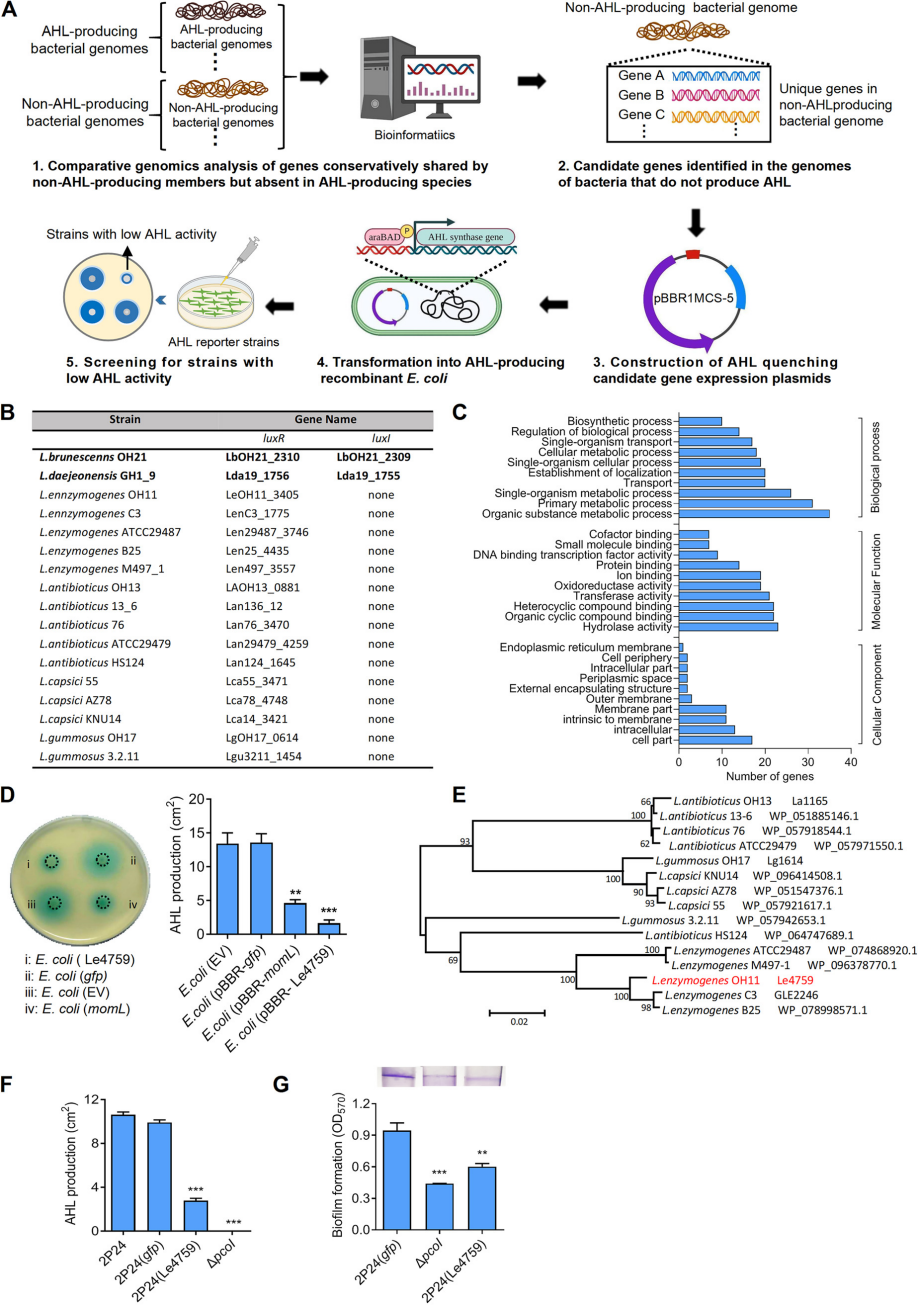
Identification of Le4759 encoding a glycosyltransferase-like protein as an AHL-quenching protein from non-AHL-producing Lysobacter enzymogenes. (A) Workflow of the defined platform for discovery of AHL-quenching protein genes from non-AHL-producing bacteria. (B) Selected genomes of AHL-producing and non-AHL-producing *Lysobacter* members. Two AHL-producing *Lysobacter* members (represented in bold) were selected, and the genomes of these 15 *Lysobacter* members do not contain LuxI but encode the so-called orphan LuxR homolog instead. (C) Go term analysis of conserved genes shared by non-AHL-producing *Lysobacter* members but absent in AHL-producing *Lyosbacter* species. (D) Ectopic expression of Le4759 encoding a glycosyltransferase-like protein in recombinant, *pcoI*-expressed E. coli significantly inhibited AHL production (left panel) and its quantification (right panel). Recombinant E. coli was induced to produce AHL using 0.1% arabinose. (E) Phylogenetic tree of Le4759 and its homologues from selected non-AHL-producing *Lysobacter* species. (F–G) Introduction of Le4759 into Pseudomonas *fluorescence* 2P24 remarkably inhibited AHL production (F) and AHL-controlled biofilm formation (G) 2P24, wild type of Pseudomonas *fluorescence*, served as a positive control; *gfp*, a negative control; Δ*pcoI*, a mutant of *P. fluorescence* 2P24 lacking the AHL synthase (PcoI) gene and served as an additional negative control. Average data from three experiments was presented, ± SD. ***, *P* < 0.0001; **, *P* < 0.01, assessed by one-way ANOVA.

While AHL QS is widespread in a wide variety of pathogenic and beneficial bacteria, several Gram-negative bacteria can block this natural system by encoding AHL-degrading enzymes, represented by AiiA from *Bacillus* sp. 240B1, MomL from Muricauda olearia, and AiiD from *Ralstonia* sp. XJ12B (20–22). These AHL-degrading enzymes are usually identified by library construction screening and are generally recognized to be canonical AHL-quenching proteins (23). Most of these canonical AHL-quenching proteins have been widely studied for application to control plant and animal pathogenic bacterial infection through AHL degradation, because quenching AHL signaling commonly results in bacteria with pathogenic defects (22, 24). Moreover, the absence of AHL synthase and presence of point-mutated, functionally inactive AHL synthases appear to be additional naturally occurring mechanisms of AHL quenching. For example, an amino acid substitution (R27S) in AHL synthase, PsyI of Pseudomonas savastanoi, results in a deficiency of PsyI in the synthesis of AHL. Frameshift mutations lead to pseudonylation of the *PsyI* gene, causing P. syringae pv. *tomato* to become a non-AHL-producing bacterium (25). The Proteobacteria genus *Lysobacter* includes many ubiquitous bacteria in the environment, and several of its members are considered promising biocontrol agents against a variety of crop fungal and/or bacterial diseases by producing abundant lytic enzymes and antimicrobial secondary metabolites (26, 27). Among them, *L. enzymogenes*, the most studied species in the genus, functions as a natural predator of fungi by producing a special antifungal antibiotic HSAF (heat-stable antifungal factor) as the major antifungal weapon (28–30). With the exception of *L. brunescens* OH21 and *L. daejeonensis* GH1_9, which possess a typical AHL synthase, almost all other reported members of *Lysobacter* did not encode the LuxI-type AHL synthase (Fig. 1B). We thus proposed that they may not produce an AHL signal (30–32).

In a recent study from our laboratory, we demonstrated that the non-AHL-producing Lysobacter enzymogenes encoded a noncanonical AHL-quenching protein termed LqqP that is identified by an immunoaffinity chromatography using the Pseudomonas *fluorescence* AHL synthase PcoI as bait. We found that LqqP fails to degrade commercial AHL *in vitro* but recognizes PcoI through direct protein-protein interaction to inhibit PcoI-dependent AHL production (32). This earlier finding prompted us to emphasize non-AHL-producing bacteria using *Lysobacter* members as a working model and hypothesize that their genomes probably encode other previously unidentified noncanonical AHL-quenching proteins.

To test the above hypothesis, we developed an efficient platform involving comparative genomics, genetic, and biochemical approaches to efficiently discover AHL-quenching proteins. Here, we provided abundant evidence to show that the non-AHL-producing members of *Lysobacter* encoded at least two previously unidentified noncanonical AHL-quenching proteins that could not degrade AHL. Our findings thus open the door for bacteriologists to identify widely distributed noncanonical AHL-quenching proteins in non-AHL-producing bacteria of interests.

## RESULTS

### Establishment of a unique platform to identify Le4759 encoding a glycosyltransferase-like protein in *Lysobacter* as a novel AHL quorum-quenching protein.

To test the hypothesis that the genomes of non-AHL-producing bacteria encode previously unidentified AHL-quenching genes/proteins, we established a novel platform involving comparative genomics, genetic screening, and biochemical studies. As shown in Fig. 1A, the platform first asked bacteriologists to carry out comparative genomic analyses between AHL-producing and non-AHL-producing members of the same genus or phylogeny. This step could result in identifying genes that are conservatively shared by non-AHL-producing members but not present in AHL-producing species. These candidate target genes were then genetically screened using recombinant AHL-producing E. coli as the host, where any AHL-synthase genes of interests could be applied. This screening facilitated the identification of positive genes that blocked AHL production in a recombinant E. coli host. AHL-quenching mechanism studies included direct AHL degradation and/or protein-protein interactions between test-positive genes and selected AHL synthases.

To test the working efficiency of the platform depicted in Fig. 1A, we selected familiar *Lysobacter* members as representative. A total of 17 genomes of different *Lysobacter* species were selected. Of these, only *L. brunescens* OH21 and *L. daejeonensis* GH1_9 were reported to produce AHL, as their genomes encoded the canonical AHL synthase (LuxI) and a cognate AHL-binding LuxR-type transcription factor (Fig. 1B). The remaining 15 selected genomes of *Lysobacter* members had no LuxI but encoded so-called orphan LuxR homologues (Fig. 1B). We attempted to search for *Lysobacter* genes/proteins that are conserved in the genomes of non-AHL-producing members but lost in the genomes of AHL-producing species. Based on this idea, we performed comparative genomic analyses on the 17 selected genomes, and finally identified 422 genes/proteins that met the screening requirements (Table S1). These genes/proteins were involved in various cellular processes and were functionally classified into 30 groups (Fig. 1C). A total of 34 genes/proteins were randomly selected from each of the 30 groups. These 34 genes were PCR cloned from *L. enzymogenes* OH11 and screened in an AHL-producing recombinant E. coli strain harboring a heterogeneous *pcoI* gene encoding an active AHL synthase from Pseudomonas *fluorescence* 2P24 (33). We attempted to find gene/protein candidates that could block AHL production when expressed in recombinant E. coli. Through such screening, we did identify Le4759 as a target encoding a potent glycosyltransferase. As shown in Fig. 1D, expression of Le4759 in recombinant E. coli significantly reduced the amounts of AHL detected by an AHL biosensor JZA1 as reported previously (34). As a positive control, *momL* encoding a known AHL-degrading enzyme (20) could efficiently reduce AHL production in recombinant E. coli. This phenomenon was not observed for the expression of the 33 remaining genes under similar test conditions (Fig. S1), which further validated the finding that Le4759 had AHL-quenching activity. Bioinformatics analyses showed that Le4759 was conserved in the genomes of 15 other non-AHL-producing *Lysobacter* members (Fig. 1E). By introducing plasmid-borne Le4759 into the native PcoI host, *P. fluorescence* 2P24 (referred to here as 2P24), we could not only observe a remarkable inhibition of AHL production (Fig. 1F, Fig. S2) but also found a significant reduction in biofilm biomass that is a known AHL-controlled phenotype in 2P24 (33) (Fig. 1G). Also, expression of Le4759 in 2P24 did not impair normal bacterial growth (Fig. S3A).

### Le4759 was unable to degrade AHL directly but recognized multiple AHL synthases through direct protein-protein interactions to block AHL production.

**FIG 2:**
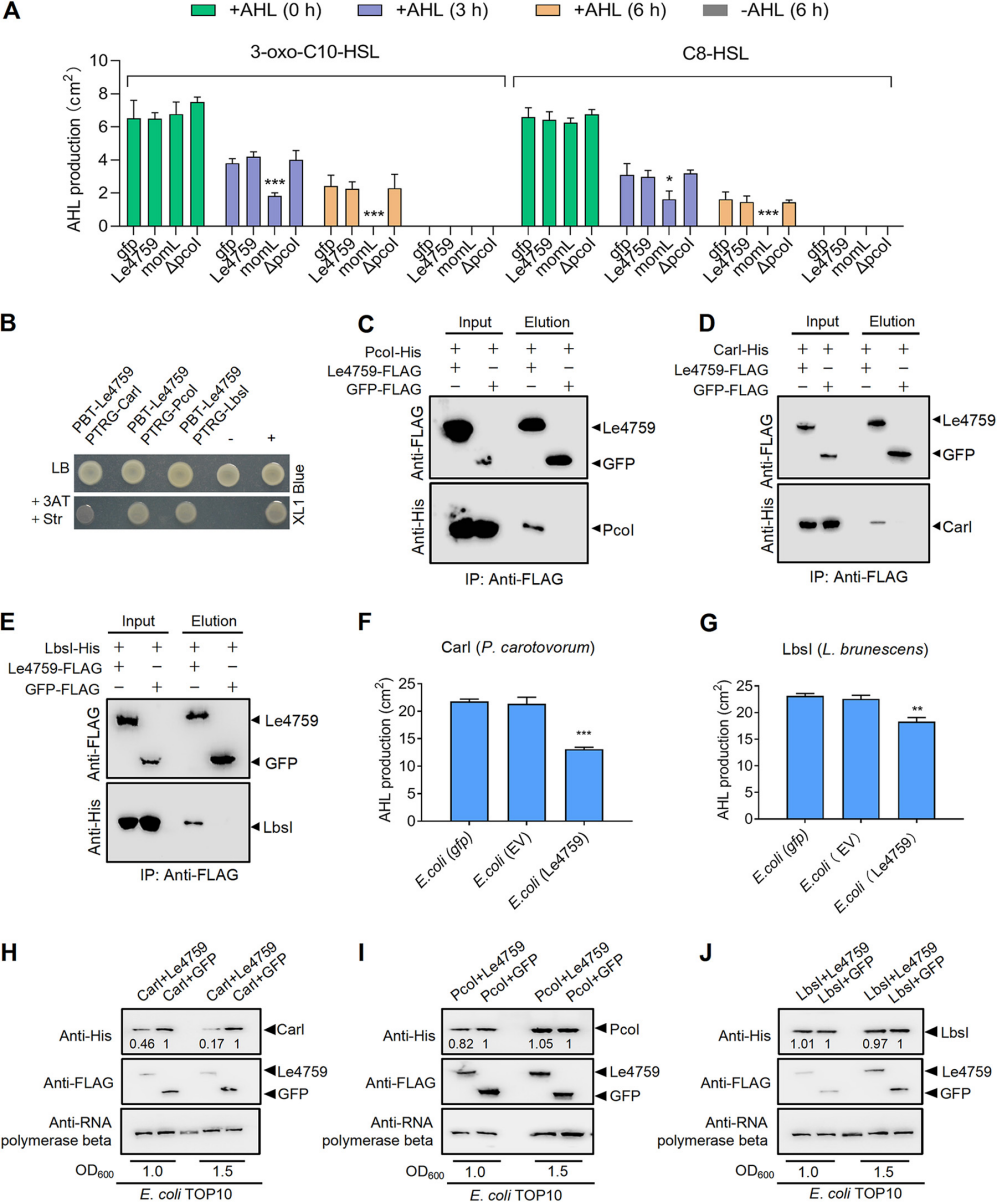
Le4759 is a noncanonical AHL-quenching protein. (A) Expression of Le4759 could not directly degrade exogenous AHL. Le4759, *gfp* or *momL* was expressed in the AHL-deficient mutant Δ*pcoI*, respectively. The commercial AHL product 3-oxo-C10-HSL was added to Δ*pcoI* cultures expressing Le4759, *gfp,* or *momL*. Degradation of AHL added after 3 and 6 h of inoculation at 28°C was determined by AHL bioassay strain JZA1. Average data from three experiments were presented, ± SD. ***, *P* < 0.0001 relative to the control, Δ*pcoI* (*gfp*)-Δ*pcoI* expresses *gfp*, assessed by one-way ANOVA. (B) Bacterial two-hybrid assay showing the interaction between Le4759 and three selected AHL synthases (PcoI, CarI, and LbsI). pTRG-PcoI/CarI/LbsI and pBT-Le4759 were coexpressed in E. coli XL1-Blue. “+,” positive control with coexpression of plasmids pBT-GacS and pTRG-GacS; “-,” negative control with coexpression of empty pTRG and pBT vectors. (C–E) Pull-down assay to confirm the interaction between PcoI-His (C), CarI-His (D), or LbsI (E) and Le4759-FLAG. Immunoprecipitation was performed using anti-FLAG antibody and Western blot was performed using anti-FLAG and anti-His antibodies. (F–G) Coexpression of Le4759 with *carI* (F) or LbsI (G) gene in E. coli significantly reduced AHL production. EV, empty vector. (H–J) Effects of Le4759 on the protein abundance of CarI (H), PcoI (I) and LbsI (J) when each of them was coexpressed with Le4759 in E. coli Top10. In panel F and G, all panels were the respective average data from three experiments. ± SD. ***, *P* < 0.0001; *, *P* < 0.05, assessed by one-way ANOVA. Band intensities were quantified and analyzed using ImageJ (https://imagej.nih.gov/ij/), with numbers representing the relative intensities of the corresponding bands. The intensity levels of the bands in lanes “CarI+GFP,” “PcoI+GFP,” or “LbsI+GFP” were set to 1.00. The beta-subunit of RNA polymerase was used as a loading control.

Next, we sought to understand the mechanism by which Le4759 achieves its AHL quorum quenching activity. We first explored whether Le4759 has the ability to directly degrade AHL, which represents a typical mode for AHL quorum quenching. For this, 100 nM commercial AHL (N-[3-oxodecanoyl]-l-homoserine lactone, 3-oxo-C10-HSL and N-octanoyl-l-homoserine lactone, C8-HSL) was added to the culture of AHL-deficient P. fluorescens mutant Δ*pcoI* expressing Le4759. The results in Fig. 2A and Fig. S4A showed that the expression of Le4759 or the negative-control *gfp* of Δ*pcoI* failed to degrade the supplemented AHL as determined by the AHL biosensor strain JZA1. Under similar conditions, expression of a positive-control *momL* encoding a known AHL-degrading enzyme (20) could efficiently degrade supplemented AHL in a time-dependent manner (Fig. 2A). These results indicate that Le4759 did not directly degrade AHL, allowing us to define Le4759 as a noncanonical AHL-quenching protein. Consistently, a BLASTP search against the NCBI database revealed no sequence similarity/identify of Le4759 with any of the reported AHL-degradation enzymes such as AiiA (Metallo-β-lactamase lactonase superfamily) (23), AttM (Metallo-β-lactamase lactonase superfamily) (35), MomL (Metallo-β-lactamase lactonase superfamily) (20), AiiM (α/β-Hydrolase superfamily) (36), GKL (Phosphotriesterase-like lactonase superfamily) (37), PONX_OCCAL (Paraoxonase family) (38), CYP102A1 (AHL oxidoreductase) (39) and AiiD (AHL acyl transferase) (21).

Next, we tested whether Le4759 blocked AHL production by targeting PcoI through direct protein-protein interaction. Via B2H, we did find that there was a direct binding between Le4759 and PcoI (Fig. 2B). This protein-protein binding was further validated by an *in vitro* pulldown assay, in which Le4759-FLAG could bind to PcoI-His (Fig. 2C), indicating that Le4759 had the ability to interact with PcoI directly. To test whether Le4759 could specifically recognize PcoI, we further selected the CarI and LbsI AHL synthases from Pectobacterium carotovorum PccS1 (herein refers to PccS1) and *L. brunescenns* OH21, respectively. To our surprise, we also found that Le4759 interacted directly with CarI or LbsI via B2H and pulldown assays (Fig. 2B and D and 2E). To investigate whether such protein-protein interactions also had biological significance, we coexpressed Le4759 or control *gfp* gene with *carI* or *lbsI* in E. coli and found that Le4759-mediated coexpression with *carI* or *lbsI* significantly reduced AHL production compared to control *gfp* gene (Fig. 2F and G, Fig. S4B & S4C). These results imply that Le4759 appears to employ a common molecular mechanism by targeting diverse AHL synthases to quench AHL quorum sensing through direct protein-protein interactions.

To understand what happens when heterogeneous AHL synthases bind to Le4759, we also selected three AHL synthases, PcoI, CarI, and LbsI for testing, as they were experimentally validated to interact directly with Le4759 as described above. We cotransformed pUCP-PcoI, pUCP-CarI or pUCP-LbsI and pBBR-Le4759 into E. coli DH5α to test whether the expression profiles of these AHL synthase genes were altered upon Le4759 binding. We found that coexpression of Le4759-*carI* in E. coli reduced the CarI expression profile at an OD_600_ of 1.0 (>2-fold) or 1.5 (>5-fold) compared to the *gfp*-*carI* control, (Fig. 2H), while the abundance of GFP or Le4759 was not affected in these coexpressions (Fig. 2H). As an internal control, the β subunit of RNA polymerase showed similar protein levels in all samples tested (Fig. 2H). Under similar testing conditions, we found that coexpression of Le4759-*pcoI* or Le4759-*lbsI* in E. coli only slightly reduced the PcoI or LbsI expression profile at an OD_600_ of 1.0 or 1.5 (Fig. 2I to J). Together, these findings uncovered that Le4759 seems to utilize distinct mechanisms to achieve AHL quenching upon binding to multiple AHL synthases, and that Le4759 most likely reduced the abundance of free CarI, thereby reducing CarI-dependent AHL production.

### Additional evidence supporting that Le4759 quenched CarI-dependent AHL signaling in *P. carotovorum*.

**FIG 3:**
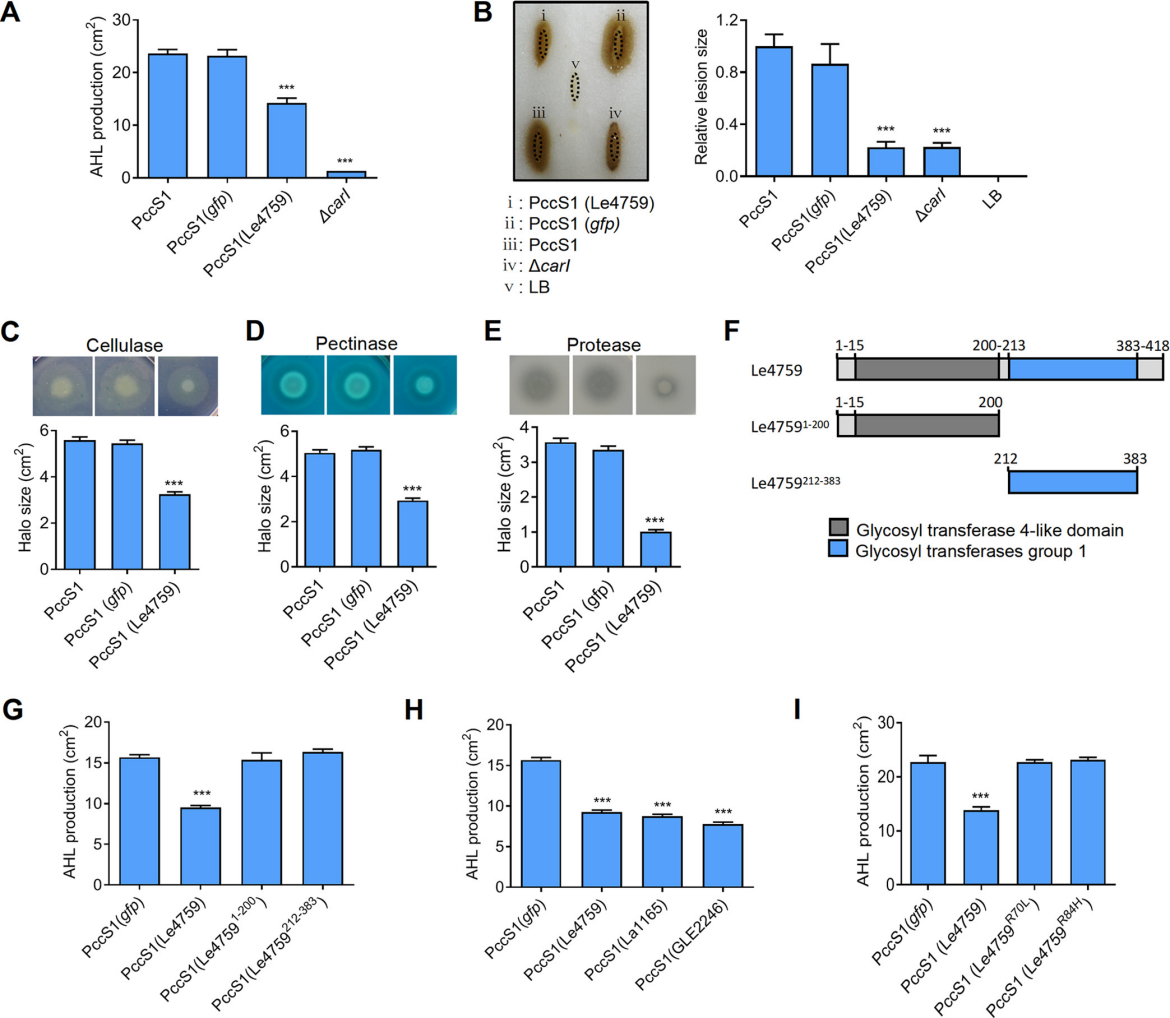
Effect of Le4759 in the CarI-encoded Pectobacterium carotovorum. (A) Introduction of Le4759 into wild-type *P. carotovorum* PccS1 significantly reduced AHL production. PccS1 expressing *gfp* and Δ*carI*, a *P. carotovorum* PccS1 mutant lacking the AHL synthase (CarI) gene, was served as a negative control. (B) Effect of Le4759 expression on the pathogenicity of PccS1. The left panel showed a representative case of disease symptoms by wild-type PccS1 and its derivatives in Chinese cabbage. The right panel was the average data from the three experiments corresponding to panel B, using the ellipse area to calculate the relative lesion size. (C–E) Effects of Le4759 expression on extracellular cellulase (C), pectinase (D), and protease (E), all of which are known AHL-controlled virulence factors. In C–E, the corresponding panels below were the average data from three experiments. (F) Scheme illustrating the prediction domain of Le4759 and its domain truncations. (G) Effect of domain truncations on Le4759-induced AHL inhibition in PccS1. (H) Effects of heterogeneous expression of two Le4759 homologous genes on AHL production in PccS1. La1165 and GLE2246 are the Le4759 homologs from the non-AHL-producing *L. antibioticus* OH13 and *L. enzymogenes* C3, respectively. (I) Identification of key amino residues required for Le4759 to function as an AHL inhibitor in PccS1. In panel G-I, all panels were the respective average data from three experiments. ± SD. ***, *P* < 0.0001, assessed by one-way ANOVA.

Since Le4759 reduced the CarI expression profile, we sought to biologically correlate this finding. For this purpose, *P. carotovorum* PccS1 encoding native CarI gene was selected. We introduced plasmid-borne Le4759 into PccS1 and observed corresponding inhibition of AHL production (Fig. 3A, Fig. S5A). Under the same nutrient conditions, we found that expression of Le4759 in PccS1 did not affect the bacterial growth ability (Fig. S3B). We next tested whether Le4759 can target CarI in PccS1 to attenuate bacterial infection and the production of multiple virulence-associated extracellular enzymes, both of which are known to be controlled by CarI-generated AHL (40). We found that mutation of *carI* completely impaired bacterial infection of the host plant Chinese cabbages, which is consistent with the previous reports (41, 42). Under similar testing conditions, we observed that PccS1 expressing Le4759 significantly reduced its ability to infect Chinese cabbages (reflected by lesion size) compared with GFP control (Fig. 3B). Furthermore, expression of Le4759 in PccS1 remarkably decreased the production of extracellular cellulase, pectinase, and proteases (Fig. 3C to E).

Next, we aimed to identify the crucial domains responsible for the functioning of Le4759 in PccS1. As shown in Fig. 3F, Le4759 possessed two predicted glycosyltransferase domains. We investigated the contribution of each domain by protein truncation. We first heterogeneously expressed the Le4759 variant gene lacking each predicted domain in PccS1, and the results clearly showed that Le4759 required both domains as a repressor of AHL synthesis in PccS1 (Fig. 3G, Fig. S5B). However, in coexpression of Le4759 and PcoI genes in E. coli where Le4759 is functional to inhibit PcoI-dependent AHL production, we detected no signal reflecting Le4759’s glycosyltransferase activity, while the positive control showed such a positive signal (Fig. S6). These results suggest that Le4759 appears not to be an active glycosyl transferase under the conditions tested. Then, we intended to identify the amino residues required for Le4759 functioning. To do this, we selected GLE2246 and La1165, which are Le4759 homologues from non-AHL-producing *L. enzymogenes* C3 and *L. antibioticus* OH13, respectively. Heterogeneous expression of GLE2246 or La1165 in PccS1, similar to Le4759, inhibited AHL production (Fig. 3H, Fig. S7). Under similar test conditions, we showed that three Le4759-unrelated, glycosyltransferase-domain containing genes (Lb2423, Pc1422, and PF5112) from Lysobacter brunescens OH21, Pectobacterium carotovorum PccS1, and Pseudomonas *fluorescence* 2P24 had no detectable AHL-quenching activity (Fig. S8). Through a sequence alignment between Le4759 and its two functionally active homologues (GLE2246 and La1165) (Fig. S9), we identified two conserved amino acid residues R70 and R84, both located in the first predicted glycosyltransferase domain (Fig. S9). We subsequently generated two Le4759 variants, Le4759^R70L^ and Le4759^R84H^, by site-directed mutagenesis. Western blotting revealed that each substitution did not affect the degree of Le4759 expression in PccS1 (Fig. S10). However, we found that PccS1 expressing each of the two Le4759 point-mutated genes was unable to inhibit AHL production (Fig. 3I, Fig. S11), suggesting that both residues were essential for the function of Le4759. Collectively, these additional genetic and phenotypic data further supported a role for Le4759 as a noncanonical AHL-quenching protein that bound to CarI to reduce its protein abundance, thereby limiting AHL production and AHL-controlled bacterial infection.

### Genes encoding noncanonical AHL-quenching proteins were common to *Lysobacter*.

**FIG 4:**
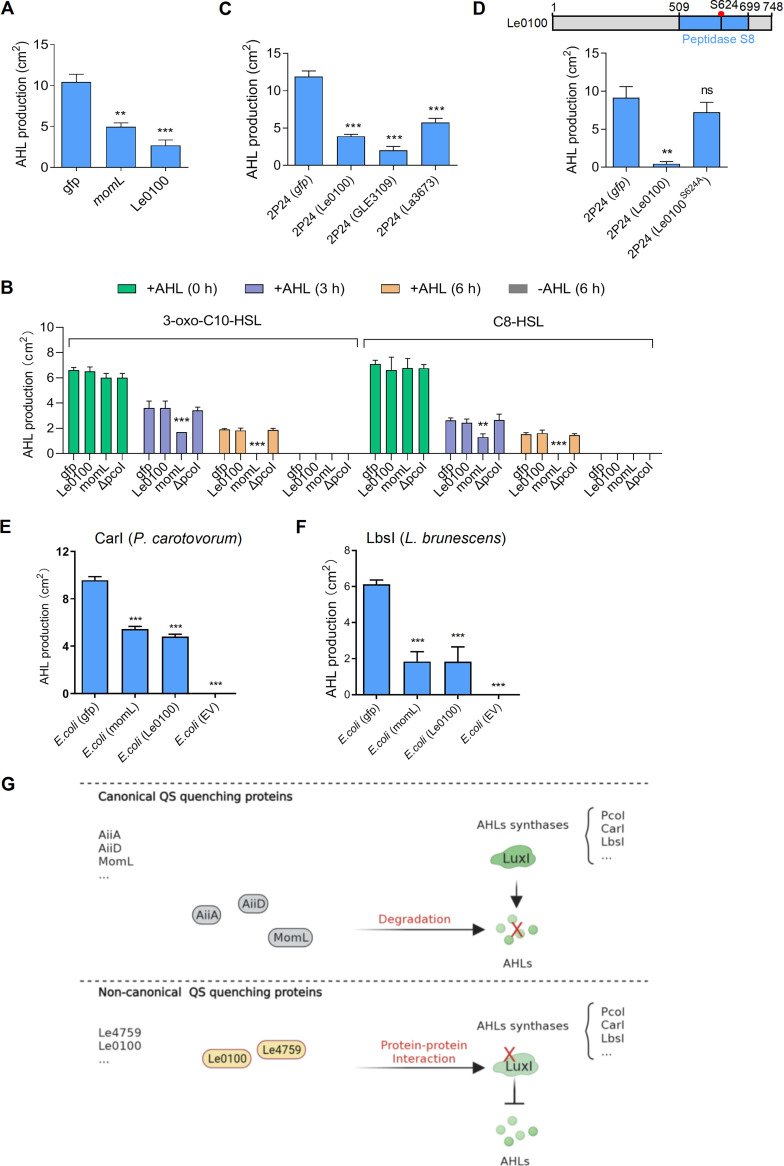
Identification of Le0100 from Lysobacter enzymogenes, which encodes a predicated peptidase as an additional noncanonical AHL-quenching protein. (A) Heterogeneous expression of *Le0100* in recombinant, *pcoI*-expressed E. coli significantly inhibited AHL production. (B) Expression of *Le0100* could not degrade AHL. This assay was carried out similar to the assay of Fig. 2A. (C) Effects of heterogeneous expression of two Le0100 homologous genes on AHL production in *pcoI*-encoded Pseudomonas *fluorescence* 2P24 (2P24). GLE3109 and La3673 are Le0100 homologs from *L. antibioticus* OH13 and *L. enzymogenes* C3, respectively. (D) Identification of the key amino residue (S624) required for Le0100 to function as an AHL-quenching protein in 2P24. In panel C and D, all panels were the respective average data from three experiments. ± SD. ***, *P* < 0.0001; **, *P* < 0.01, assessed by one-way ANOVA. ns, no statistical significance. (E–F) Coexpression of *Le0100* with *carI* (E) or *lbsI* (F) gene in E. coli significantly reduced AHL production. EV, empty vector. (G) A proposed model accounting for the different modes of action of those well-characterized canonical AHL-degrading enzymes and noncanonical AHL-quenching proteins presented in this study. Canonical AHL-quenching proteins of AiiA from *Bacillus* sp. 240B1, MomL from Muricauda olearia, and AiiD from *Ralstonia* sp. XJ12B could degrade AHLs produced by various AHL synthases listed in the right. To achieve this, these canonical AHL-quenching proteins do not need to interact with AHL synthases. In contrast, the noncanonical AHL quorum-quenching protein presented in this study could not degrade AHLs, whereas they, represented by Le4759, could bind diverse AHL synthases through direct protein-protein interactions, thereby blocking AHL production.

The discovery of Le4759 as a noncanonical AHL-quenching protein prompted us to explore whether it is a common issue in *Lysobacter*. For this purpose, we randomly screened 14 additional genes (Fig. S12 and Table S1) that are absent in the two available AHL-producing *Lysobacter* species but are conservatively encoded by the genomes of the 15 remaining non-AHL-producing *Lysobacter* members, as shown in Fig. 1B. Via this step, we did identify Le0100, which encodes a predicated peptidase as a second noncanonical AHL-quenching protein. Le0100 strongly inhibited the PcoI-dependent AHL production in E. coli, and it also failed to degrade exogenous AHL of C8-HSL and 3-oxo-C10-HSL (Fig. 4A and B; Fig. S13A and B). We did observe that Le0100 expression in 2P24 did not affect bacterial growth (Fig. S3C). Le0100 seems to be conserved among members of the genus *Lysobacter* that did not produce AHL (Fig. S14). Two Le0100 homologs (GLE3109 and La3673) from *L. enzymogenes* C3 (GLE3109) and *L. antibioticus* OH13 (La3673), respectively, also exhibited the ability to inhibit PcoI-dependent AHL production when each of them was heterogeneously expressed in *P. fluorescence* 2P24 (Fig. 4C, Fig. S15A). In addition, when we submitted the amino sequence of Le4759 and Le0100 into NCBI database to search their respective homologues in non-*Lysobacter* genomes, we found that the homologues of Le4759 and Le0100 were also distributed in several bacterial genomes with or without LuxI-type AHL synthase; however, the homologues of Le4759 and Le0100 were absent in the AHL-producing strains Pseudomonas *fluorescence* 2P24 and Pectobacterium carotovorum PccS1 (Table S2). We further found the conserved S624 amino acid residue within the peptidase domain was required for Le0100 to function as a noncanonical AHL-quenching protein (Fig. 4D; Fig. S15B and S16). To further support this, we coexpressed the Le0100 or control *gfp* gene with *carI* or *lbsI* in E. coli and found that Le0100-mediated coexpression with *carI* or *lbsI* significantly reduced AHL production compared to control *gfp* gene (Fig. 4E and F; Fig. S17A and B). These findings revealed that genes encoding atypical AHL-quenching protein appear to be a common event in non-AHL-producing *Lysobacter* and further supported the power of the developed platform to discover novel noncanonical AHL-quenching proteins.

## DISCUSSION

Over the past few decades, various AHL-degrading enzymes have been identified from numerous bacterial species, which are considered to be canonical AHL-quenching proteins that efficiently degrade diffusible AHLs produced by discrete AHL synthases to quench their quorum sensing capabilities (22, 43). Therefore, AHL-degrading enzymes have been developed as effective means of controlling bacterial diseases, as AHL quenching often results in bacteria with pathogenic defects (44–46). In this study, we designed a novel platform that enabled the discovery of two noncanonical AHL-quenching proteins, Le4759 and Le0100. They are both non-AHL-degrading enzymes, and Le4759 could block AHL generation by recognizing multiple AHL synthases via direct protein-protein interactions. If we analogize AHL signaling and AHL synthase as AHL-targeting “weapons” and “arsenals,” respectively, our findings suggest that Le4759 preferred to targeting AHL “arsenals” rather than those known AHL-degrading enzymes that prefer to target AHL “weapons.” Thus, our findings provided an unusual mode of action for regulating AHL quorum quenching (Fig. 4G). At the application level, although AHL synthase-binding quorum-quenching proteins from non-AHL producing bacteria (such as Le4759 and Le0100) do not appear to have a good chance of entering directly into the cells of AHL-producing pathogens to block their AHL production and suppress AHL-controlled infection, this can be achieved artificially through nanotechnology that is a promising approach to facilitate delivery of materials of interest into target cells (47, 48). Therefore, we proposed that a noncanonical quorum-quenching protein (i.e., Le4759) could be developed as a promising next-generation anti-infective drug to overcome emerging bacterial antibiotic resistance.

The findings presented in this study also outlined previously uncharacterized clues to explain the natural loss of widespread AHL quorum sensing during bacterial genome evolution (25). Previously, bacteria that do not produce AHL are often explained by production of AHL-degrading enzymes, lack of AHL synthases, and the presence of functionally inactive AHL synthases through natural point mutation (25). Unlike these earlier findings, we found an additional mechanism to explain this natural phenomenon. We thought that members of the *Lysobacter*, which do not produce AHL, could evolutionarily retain non-AHL-degrading proteins represented by Le4759 to recognize and target different AHL synthases through protein-protein interactions. This unusual ability enabled Le4759 to target certain AHL synthases such as CarI to reduce their free abundance or destabilize them, thereby inhibiting their ability to produce AHL. Since Le4759 is conservatively retained and the AHL synthase was selectively “deleted” during the genome evolution of non-AHL-producing *Lysobacter*, we proposed the genomic presence of non-AHL-degrading proteins with quorum-quenching activity as a naturally occurring mechanism to drive the loss of the AHL synthases. As supporting evidence, we did recently identify *L. enzymogenes* LqqP as a natural intracellular AHL inhibitor protein (32).

The recognition of multiple AHL synthases by Le4759 is notable because it suggests that artificial delivery of this noncanonical AHL-quenching protein into a variety of AHL-producing pathogenic bacterial cells is an alternative option for controlling bacterial infection in crop protection and/or medical therapy. In support of this, we demonstrated that heterogeneous expression of Le4759 in phytopathogenic PccS1 significantly attenuated its virulence to the host plant. Although we currently do not know the structural basis for the recognition of multiple AHL synthases by Le4759 at the mechanistic level, we at least demonstrated that its binding to CarI, the AHL synthase of PccS1, reduced its protein abundance, although the underlying mechanism still remains unknown. However, it is possible that binding CarI by Le4759 probably altered the CarI conformational state, causing it to become an aberrant protein in E. coli. In this case, the E. coli Lon protease, an ATP-dependent serine protease that mediates selective degradation of protein variants or abnormal proteins, may be involved in this process (49). In addition, although Le4759 did not exploit this mechanism to block PcoI/LbsI-dependent AHL production, binding of Le4759 to PcoI/LbsI may interfere with the ability of these two AHL synthases to recognize or bind substrates required for *de novo* AHL synthesis.

It is also noteworthy that Le4759 was predicted as a glycosyltransferase, and we found that each glycosyltransferase domain was essential for Le4759 to function as a noncanonical AHL-quenching protein. However, under the *in vitro* conditions, we did not detect any glycosyltransferase activity (Fig. S6). This outlined at least two possibilities: one is that Le4759 may act as an active glycosyltransferase *in vivo*, but requiring unidentified cofactors, and the other is that Le4759 is not an active glycosyltransferase despite its predicted glycosyltransferase domains. Even Le4759 maybe a *bona fide* glycosyltransferase, its function may be independent of its enzymatic activity. This has been demonstrated in our laboratory, where we found that LqqP functions as an intracellular AHL inhibitor protein independent of aminopeptidase activity (32). We are now elucidating the protein structure of Le4759 to answer this question.

### Conclusion.

Since the noncanonical AHL-quenching proteins Le4759 and Le0100 could recognize diverse AHL synthases to block their ability to produce AHL, we defined them as previously unidentified inhibitors of intracellular AHL production. These striking features might be one of the intrinsic reasons to explain the natural loss of AHL synthases during the genomic evolution of non-AHL-producing *Lysobacter*, because these intracellular AHL inhibitors represented by Le4579 gained binding to certain AHL synthases (i.e., CarI) to destabilize them or reduce their free abundance. We believe that the designed platform was powerful and versatile, allowing bacteriologists to discover other previously unidentified noncanonical AHL quenching proteins.

## MATERIALS AND METHODS

### Bacterial strains, plasmids, and growth conditions.

Table S3 lists the strains and plasmids used in this study. Different Escherichia coli strains were grown in Lysogeny broth (LB) medium at 37°C with the addition of appropriate antibiotics. Unless otherwise stated, the following antibiotics were used to cultivate Lysobacter enzymogenes, *P. carotovorum* PccS1, and Pseudomonas fluorescens 2P24: kanamycin (Km), gentamicin (Gm), and tetracycline (Tc) for plasmid maintenance. *P. carotovorum* PccS1, Pseudomonas fluorescens 2P24, and their derivatives were cultured in liquid LB medium at 28°C, and their growth was monitored by measuring the optical density at 600 nm (OD_600_) every 2 h. Each strain was performed three times and the average OD_600_ values were used to generate bacterial growth curves.

### Bioinformatics analyses.

The genomes of 17 representative *Lysobacter* species were obtained from the GenBank database. To identify the presence of LuxI-type AHL synthase in selected bacterial genomes, local BLASTp was run using LuxI (GenBank: AAP22376.1) and LuxR (GenBank: CAA68561.1) as queries. When the E value was lower than 10^−5^, it was considered that LuxR-type AHL synthase and LuxR-type transcription factor were present. The similarity to the corresponding homologous protein was usually higher than 40%. Sequence alignment was then performed to find genes shared by 15 *Lysobacter* strains without AHL synthetase (LuxI), but absent in the two *Lysobacter* species with AHL synthase (LuxI). To search for conserved genes that coexist only in non-AHL-producing *Lysobacter* species, *L. ennzymogenes* OH11 was selected as a reference. The entire genome of *L. ennzymogenes* OH11 was used as a blast query against other tested *Lysobacter* species. Similar genes/proteins were considered to be present when the E value is lower than 10^−5^. By this step, 422 proteins from OH11 met the established criteria that were conserved in the genomes of non-AHL-producing members but absent in the genomes of AHL-producing species.

To identify homologous proteins to Le4759 and Le0100, we ran local BLASTp using either Le4759 or Le0100 as the query to identify the corresponding homology in selected bacterial genomes. When the E value is lower than 10^−5^, it was considered that there was a similar protein, and the similarity percentage with the corresponding homologous proteins, is usually higher than 40%.

### Heterogeneous expression of AHL synthetase gene in E. coli.

The coding regions of PcoI from P. fluorescens 2P24, *carI* from *P. carotovorum* PccS1, and *lbsI* from *L. brunescenns* OH21 were amplified by PCR using the primers given in Table S4. Restrictive enzyme digestion was used to clone PCR product into the broad host vectors pBBR1-MCS5 and pUCP26 (Table S3). Plasmid integrity was confirmed by PCR and Western blotting by electroporating the resultant plasmid into the DH5α strains of E. coli.

### AHL bioassays.

The AHL plate bioassay was carried out as previously mentioned (34). Agrobacterium tumefaciens JZA1 is an efficient AHL bioassay strain for identification of AHL production (34). The JZA1 strain was inoculated in 100 mL of LB liquid medium with final antibiotic concentrations of gentamicin 30 μg/mL, kanamycin 30 μg/mL, spectinomycin 30 μg/mL, and tetracycline 12 μg/mL, and incubated at 28°C for 24 h until the OD_600_ reached 1.5. We then added 10 mL of JZA1 culture to 50 mL of solid LB medium at 50°C with the addition of 5-bromo-4-chloro-3-indolyl-d-galactoside (X-gal) to a final concentration of 40 g/mL. Plates containing JZA1 were prepared after mixing, and then 3 μL of AHL samples were seeded on the surface of the plates. The prepared dishes were incubated at 28°C for 18 h, and the size of the blue circle was observed.

### Bacterial two-hybrid assay.

Protein-protein interactions were discovered using the BacterioMatch II two-hybrid (B2H) system (Agilent Technologies, USA) as previously mentioned (50). In brief, the coding region of Le4759 target protein was cloned into the pBT plasmids, and the coding regions of PcoI, CarI and LbsI target proteins were cloned into the pTRG plasmid, and then transformed into the blue MRF Kan strain of E. coli. Positive controls included plasmids pBT-GacS and pTRG-GacS (Table S3), while negative controls included transformants with empty pTRG and pBT vectors. Selective medium was spotted onto each cotransformant and cultivated at 28°C for 2 days. If the two proteins physically interact, transformed E. coli strain carrying both vectors should thrive on reference medium (+3AT+Str^r^) based on minimal medium (M9) supplemented with 5 mM 3-AT, 2 μg/mL Str, 12.5 μg/mL tetracycline, 34 μg/mL chloramphenicol, and 30 μg/mL kanamycin as described in previous work (50). As previously mentioned, LB agar is a nonselective medium (-3AT-Str^r^) that includes 12.5 μg/mL tetracycline, 34 μg/mL chloramphenicol, and 30 μg/mL kanamycin (50). This medium was designed to ensure successful transformation of both vectors into E. coli blue MRF Kan.

### Pull-down and Co-IP assays.

Pull-down assays were performed as previously described (51). The reaction mixture contains 5 μM His-tagged protein and FLAG-tagged protein in 1.5 mL of PBS buffer. 50 μL of anti-His magnetic beads (Bimake, Shanghai, China) were then added and the mixture incubated overnight at 4°C. Magnetic beads were collected at 4°C and washed 3 times with PBS containing 1% Triton X-100 to remove nonspecifically bound proteins. Proteins captured on FLAG-beads were eluted by boiling with 4×SDS loading dye for 10 min, followed by SDS-PAGE and Western blotting.

The Co-IP assays were performed as in a previous work (51). Briefly, the coding regions of the target proteins (Le4759, PcoI, CarI, and LbsI) were cloned into pBBR1-MCS5 and pUCP26 plasmids and cotransformed into E. coli DH5α. Transformants were grown in LB until OD_600_ reached 1.5. 40 mL of cultured cells were collected, then resuspended in 2 mL of 0.1 M PBS (pH, 7.4), and sonicated (Sonifier 250; Branson Digital Sonifier, Danbury, USA). It was centrifuged at 6,000 rpm for 20 min at 4°C to remove insoluble material. Fifty all of anti-FLAG magnetic beads (Bimake, Shanghai, China) were added and incubated overnight at 4°C. The elution procedure was consistent with the pulldown assays. Protein detection involved the use of FLAG-(M20008S) and His-(ab18184) specific antibodies (Abmart, Shanghai, China).

### Biofilm formation assays.

Biofilm formation by P. fluorescens 2P24 was carried out as previously described (33). In summary, P. fluorescens 2P24 and its derivative strains were grown in liquid LB until OD_600_ reached 1.0. 500 μL of bacterial culture was then taken, added to a 2-mL tube containing 1 mL of fresh LB, and incubated at 28°C for 36 h. Biofilms were washed three times with sterilized ultrapure water, treated with 0.3% crystal violet (CV) for 15 min, then washed three times again with ultrapure water and resuspended in 95% ethanol (1 mL). Biomass was quantified at 570 nm with a spectrophotometer (Biotek, USA).

### Virulence assays.

Plant virulence assays on leaves were performed as previously described (52, 53). Briefly, PccS1 and its derivative strains were grown overnight at 28°C. Cells were harvested and suspended in sterilized water to an OD_600_ of 1.0 (10^8^ CFU mL^−1^) for infection assays. About 45-day-old leaves were removed from Chinese cabbage. The isolated leaves were surface sterilized with 75% ethanol. The backs of the leaves at each site were pierced with a sterilized toothpick, and 10 μL of bacterial suspension was applied in parallel on the wound. Sterile water was inoculated as a negative control. The leaves were incubated in a humid chamber at 28°C, and the immersed area of the leaves was measured 16 h after inoculation. Toxicity assays were repeated three times independently.

Exoenzyme activity assays were performed as previously described (54). Briefly, for the pectinase assays, 2 μL of the bacterial strain to be tested was dropped on the surface of the pectinase detection plate (1.6% [w/v] Bacto agar, 0.1% (w/v) yeast extract, 0.1% (w/v) [NH_4_])_2_SO_4_, 1 mM MgSO_4_, 0.5% [v/v] glycerol, 0.5% [w/v] polygalacturonic acid, [sodium salt], 20% [v/v] pel phosphate buffer [15 g Na_2_HPO_4_, 0.7 g NaH_2_PO_4_▪H_2_O per litre, pH 8.0], cultured at 28°C for 48 h, and stained with 7.5% (W/V) copper acetate solution for 1 h to observe the double-layered opalescent degradation circle on a blue background. To detect cellulase activity, 2 μL of bacterial droplets of the strain to be tested were placed on the surface of the enzyme detection plate (1.6% [wt/vol] Bacto agar, 1% [wt/vol] carboxymethylcellulose, 0.5% [wt/vol]) yeast extract, 0.2% [vol/vol] glycerol, 2% [vol/vol] 50× phosphate buffer [350 g K_2_HPO_4_, 100 g KH_2_PO_4_, pH 6.9], 0.1% [wt/vol] [NH4]_2_SO_4_, 0.01% [wt/vol] MgSO_4_, cultured at 28°C for 48 h, stained with 0.1% (W/V) Congo red for 20 min, and then decolorized with 1 M NaCl solution for 15 min. The decolorized solution was then poured out, and counterstained with 1 M HCl solution for 5 min to observe the gray-white degradation circles against a light blue background. For protease activity assays, 2 μL of the strain to be tested was placed on the surface of the protease detection plate (Oxoid nutrient agar, supplemented with 0.03% [wt/vol] gelatin) and cultured at 28°C for 48 h. The size of the transparent degradation circle was then observed. All tests were repeated 3 times independently.

### Data availability statement.

The sequence data from the present study have been submitted to the NCBI GenBank under the following accession numbers: OP292675 (Le4759) and OP292676 (Le0100). The genomes of *Lysobacter* species used in this study could be found in the NCBI GenBank under the following accession numbers: GCA_000768355.1, GCA_003789105.1, GCA_001442515.1, GCA_900106525.1, GCA_002024615.1, GCA_002355975.1, GCA_000731095.1, GCA_001442745.1, GCA_001442535.1, GCA_000336385.3, GCA_001442785.1, GCA_000604005.2, GCA_002355295.1, and GCA_001442805.1.
