# Supplementary material for: A Novel and Efficient Platform for Discovering Noncanonical Quorum-Quenching Proteins
Source: Microbiol Spectr. 2022 Dec 7;11(1):e03437-22. doi: 10.1128/spectrum.03437-22 (PMC9927378; doi:10.1128/spectrum.03437-22)
Supplement: Supplemental file 1 — Supplemental material. Download spectrum.03437-22-s0001.pdf, PDF file, 3.4 MB [file spectrum.03437-22-s0001.pdf]

Supplementary Information

A novel and efficient platform for discovering non-canonical quorum-quenching proteins

Jinxing Liao[a], Zihan Li[a], Dan Xiong[a], Danyu Shen[a], Lu Wang[b], Xiaolong Shao[a], Tao Li[c]*, Guoliang Qian[a*]

This file contains 4 supplemental tables and 17 supplemental figures.

Table S1 Gene candidates identified in *L. enzymogenes* for quenching AHL quorum sensing

Gene ID	Functional annotation
Le0007	heparinase III protein [Pedobacter heparinus]
Le0012	hypothetical protein [Rudaea cellulosilytica]
Le0022	NA
Le0040	NA
Le0060	hypothetical protein [Dyella japonica]
Le0081	Muramidase-2 [Stenotrophomonas maltophilia]
Le0095	hypothetical protein [Variovorax paradoxus EPS, Varpa_3397]
Le0096	low temperature requirement A protein LtrA [Cellvibrio sp. BR]
Le0100	NA
Le0101	hypothetical protein [Dyella japonica]
Le0138	hypothetical protein [Xanthomonas axonopodis]
Le0139	hypothetical protein [Chondromyces apiculatus|Myxococcus sp.]
Le0141	hypothetical protein [Xanthomonas sp. SHU308]
Le0144	putative membrane protein [Pseudomonas fluorescens SBW25]
Le0172	hypothetical protein [Rhodanobacter thiooxydans]
Le0178	NA
Le0190	GCN5 family acetyltransferase [Singulisphaera acidiphila]
Le0194	NA
Le0213	conserved hypothetical protein [Azotobacter vinelandii]
Le0215	hypothetical protein [Pseudomonas aeruginosa]
Le0239	pilus assembly protein [Stenotrophomonas maltophilia]
Le0273	NA
Le0284	pilus assembly protein [Stenotrophomonas maltophilia]
Le0297	peptide transporter [Singularimonas variicoloris]
Le0303	hypothetical protein [Stenotrophomonas maltophilia]
Le0307	unnamed protein product [Photorhabdus luminescens subsp. laumondii TTO1]
Le0311	glycosyl hydrolase [Stenotrophomonas maltophilia]
Le0315	DeoR family transcriptional regulator [Xanthomonas sp. SHU199]
Le0345	NA
Le0387	pyrroloquinoline quinone biosynthesis protein PqqB [Xanthomonas vesicatoria]
Le0388	pyrroloquinoline quinone biosynthesis protein PqqC [Xanthomonas campestris]
Le0389	coenzyme PQQ biosynthesis protein D [Xanthomonas sp. SHU308]
Le0525	Alkyl hydroperoxide reductase AhpD [Wohlfahrtiimonas chitiniclastica]
Le0533	protein of unknown function DUF6 transmembrane [Haliangium ochraceum DSM 14365]
Le0612	hypothetical protein [Stenotrophomonas maltophilia]
Le0622	sugar transporter [Burkholderia pyrrocinia]
Le0623	hypothetical protein [Dyella ginsengisoli]
Le0627	hypothetical protein [Variovorax paradoxus]
Le0633	NA
Le0661	hypothetical protein [Corallococcus coralloides]
Le0662	hypothetical protein [Corallococcus coralloides]

Le0695	hypothetical protein [Achromobacter piechaudii]
Le0748	aspartyl-asparaginyl beta-hydroxylase [Xanthomonas axonopodis]
Le0753	hypothetical protein [Alcanivorax dieselolei]
Le0754	NA
Le0797	CAAX amino terminal protease [Burkholderia sp. CCGE1002]
Le0840	Acetyltransferase, GNAT family protein [Polymorphum gilvum SL003B-26A1]
Le0866	hypothetical protein [Arhodomonas aquaeolei]
Le0873	NA
Le0915	Beta-lactamase [Azospirillum lipoferum]
Le0923	hypothetical protein [Pseudomonas geniculata]
Le0926	hypothetical protein [Stenotrophomonas maltophilia]
Le0927	lanthionine synthetase C-like protein [Lysobacter sp. ATCC 53042]
Le0969	GCN5-related N-acetyltransferase [Pseudomonas mendocina NK-01]
Le0982	NA
Le1035	acetyltransferase [Leptolyngbya sp. PCC 7375]
Le1036	integral membrane-like protein [Pseudoxanthomonas suwonensis 11-1]
Le1044	NA
Le1066	NA
Le1071	hypothetical protein [Pseudomonas mandelii]
Le1072	aerobic-type carbon monoxide dehydrogenase, large subunit CoxL/CutL-like protein [Pseudomonas sp. GM79]
Le1073	(2Fe-2S)-binding protein [Pseudomonas fluorescens]
Le1074	XshC-Cox1-family protein [Stenotrophomonas maltophilia]
Le1075	CTP: molybdopterin cytidylyltransferase [Stenotrophomonas maltophilia]
Le1077	NA
Le1079	tetracenomycin polyketide synthesis O-methyltransferase TcmP [Streptomyces himastatinicus ATCC 53653]
Le1083	Lysine exporter protein (LYSE/YGGA) [Ralstonia pickettii 12D]
Le1092	propeptide, PepSY amd peptidase M4 [Nitrobacter sp. Nb-311A]
Le1093	NA
Le1097	D-alanyl-D-alanine dipeptidase [Rhodanobacter sp. 2APBS1]
Le1100	transglutaminase [Pseudoxanthomonas sp. GW2]
Le1111	oxidoreductase [Xanthomonas campestris]
Le1112	putative ferredoxin protein [Xanthomonas albilineans GPE PC73]
Le1116	Uncharacterized metalloprotease [Stenotrophomonas maltophilia]
Le1120	hypothetical protein [Leeia oryzae]
Le1122	hypothetical protein [Leeia oryzae]
Le1124	NA
Le1161	putative transcriptional regulator [Xanthomonas sacchari]
Le1164	hypothetical protein [Burkholderia sp.]
Le1168	putative RNA polymerase sigma factor [Xanthomonas sacchari]
Le1181	conserved hypothetical protein [Xanthomonas campestris]
Le1195	major facilitator superfamily protein [Rhodanobacter fulvus Jip2]

Le1196	NA
Le1198	family 2 glycosyl transferase [Granulicella mallensis]
Le1199	hypothetical protein [Xanthomonas axonopodis]
Le1202	NA
Le1204	NA
Le1205	N-acetylmuramyl-L-alanine amidase, negative regulator of AmpC, AmpD [Niastella koreensis]
Le1206	NA
Le1208	NA
Le1210	3-carboxymuconate cyclase [Pseudomonas sp. GM17]
Le1222	hypothetical protein [Rhodanobacter spathiphylli]
Le1273	ABC transporter substrate-binding protein [Stenotrophomonas maltophilia]
Le1305	conserved hypothetical protein [Methylococcus capsulatus]
Le1319	NA
Le1321	hypothetical protein HMPREF0995_05895, partial [Lachnospiraceae bacterium 7_1_58FAA]
Le1372	cupin domain-containing protein [Rhodanobacter sp. 116-2]
Le1373	ArsR family transcriptional regulator [Burkholderia sp. JPY251]
Le1378	NA
Le1379	hypothetical protein [Variovorax paradoxus]
Le1383	putative esterase of the alpha-beta hydrolase superfamily [Deinococcus peraridilitoris DSM 19664]
Le1388	putative protease [Pectobacterium carotovorum subsp. carotovorum PCC21]
Le1391	4-oxalocrotonate tautomerase [Achromobacter piechaudii]
Le1394	hypothetical protein [Rudaea cellulosilytica]
Le1421	GCN5-related N-acetyltransferase [Thioalkalivibrio sulfidophilus HL-EbGr7]
Le1422	NA
Le1427	hypothetical protein [Rudaea cellulosilytica]
Le1458	YadA domain protein [Burkholderia ambifaria MEX-5]
Le1463	NA
Le1495	hypothetical protein [Streptomyces somaliensis]
Le1498	hypothetical protein [Streptomyces sp. FxanaD5]
Le1500	NA
Le1501	Protein of unknown function (DUF3526) [Novosphingobium sp. AP12]
Le1504	protein with peptidoglycan-binding domain [Psychroflexus torquis]
Le1505	hypothetical protein [Myxococcus sp. (contaminant ex DSM 436), A176_1024]
Le1540	hypothetical protein CATMIT_01824, partial [Catenibacterium mitsuokai DSM 15897]
Le1544	NA
Le1558	methyltransferase [Stenotrophomonas maltophilia]
Le1566	putative glycosyl transferase PslI [Pseudomonas fluorescens A506]
Le1567	NA
Le1568	arabinose efflux permease family protein [Phyllobacterium sp. YR531]
Le1570	NA
Le1574	unknown [Lysobacter enzymogenes]
Le1575	unknown [Lysobacter enzymogenes]

Le1577	SctL [Lysobacter enzymogenes]
Le1578	NA
Le1579	unknown [Lysobacter enzymogenes]
Le1581	unknown [Lysobacter enzymogenes]
Le1585	PhaA [Lysobacter enzymogenes]
Le1589	NA
Le1591	type III secretion protein, HrpO family [Burkholderiales bacterium JOSHI_001]
Le1594	NA
Le1595	hypothetical protein [gamma proteobacterium]
Le1614	NA
Le1615	NA
Le1652	relaxation protein [Xanthomonas campestris]
Le1675	transmembrane anti-sigma factor [Pseudomonas fluorescens]
Le1680	putative protease with the C-terminal PDZ domain [Pseudoxanthomonas spadix BD-a59]
Le1682	probable glucans biosynthesis protein d precursor [Xanthomonas albilineans GPE PC73]
Le1689	hypothetical protein [Dyella ginsengisoli]
Le1691	hypothetical protein [Dyella ginsengisoli]
Le1694	hypothetical protein [Rudaea cellulosilytica]
Le1695	hypothetical protein [Rudaea cellulosilytica]
Le1702	NA
Le1719	NA
Le1723	NA
Le1724	hypothetical protein [Stenotrophomonas maltophilia]
Le1740	pyridine nucleotide-disulfide oxidoreductase [Pseudoxanthomonas suwonensis]
Le1750	NA
Le1753	hypothetical protein [Enterobacter cloacae]
Le1760	peptidase C39 [Burkholderia pyrrocinia]
Le1770	NA
Le1792	putative metal-dependent membrane protease [Xanthomonas axonopodis pv. citrumelo F1]
Le1806	2, 5-diketo-D-gluconic acid reductase [Variovorax paradoxus]
Le1810	hypothetical protein [Delftia acidovorans]
Le1822	NA
Le1823	NA
Le1837	NA
Le1869	putative integron gene cassette protein [uncultured bacterium]
Le1870	streptomycin 3"-phosphotransferase [Achromobacter xylosoxidans C54]
Le1922	NA
Le1965	endoribonuclease L-PSP [Burkholderia multivorans]
Le1966	short chain fatty acid transporter [Stenotrophomonas maltophilia]
Le1982	NA
Le1991	heat shock protein Hsp70 [Xanthomonas gardneri]
Le1999	hypothetical protein [Xanthomonas vesicatoria]
Le2006	conserved hypothetical protein [Xanthomonas campestris]

Le2013	NA
Le2014	NA
Le2015	NA
Le2017	NA
Le2029	RDD family protein [Cesiribacter andamanensis]
Le2048	kanamycin nucleotidyltransferase, partial [Catenibacterium mitsuokai DSM 15897]
Le2071	NA
Le2109	NA
Le2143	hypothetical protein [uncultured bacterium, ACD_23C00190G0001]
Le2145	NA
Le2156	GCN5 family acetyltransferase [Rhodanobacter fulvus]
Le2179	conserved hypothetical protein [Ralstonia syzygii R24]
Le2180	hypothetical protein [Dyella ginsengisoli]
Le2185	NA
Le2196	membrane protein [Xanthomonas sacchari]
Le2198	NA
Le2199	hypothetical protein [Gordonia polyisoprenivorans]
Le2260	polymerase [Stenotrophomonas maltophilia]
Le2270	N-acylglucosamine 2-epimerase [Stenotrophomonas maltophilia]
Le2274	sugar hydrolase [Pseudomonas geniculata]
Le2286	conserved hypothetical protein [Stigmatella aurantiaca]
Le2288	hypothetical protein [Acidovorax citrulli]
Le2359	putative iron transport/utilization related protein [Burkholderia xenovorans LB400]
Le2407	glyoxalase/bleomycin resistance protein/dioxygenase [Rhodococcus triatomae]
Le2417	TetR family transcriptional regulator [Rhizobium leguminosarum]
Le2427	trans-2-enoyl-CoA reductase [Xanthomonas campestris]
Le2450	lanthionine synthetase [Stenotrophomonas maltophilia]
Le2521	hypothetical protein [Herbaspirillum sp. JC206]
Le2530	hypothetical protein [Stenotrophomonas maltophilia]
Le2531	hypothetical protein [Xanthomonas sacchari]
Le2540	hypothetical protein [Bifidobacterium adolescentis]
Le2552	hypothetical protein [Pseudoxanthomonas sp. GW2]
Le2572	NA
Le2616	hypothetical protein [Rudaea cellulosilytica]
Le2620	NA
Le2627	hypothetical protein, partial [Xanthomonas sp. SHU199]
Le2636	conserved hypothetical exported protein [Stenotrophomonas maltophilia K279a]
Le2649	membrane protein [Stenotrophomonas maltophilia]
Le2660	hypothetical protein [Micromonospora sp.]
Le2683	glycerol kinase [Xanthomonas sp. SHU199]
Le2685	glycerol-3-phosphate dehydrogenase [Rhodanobacter sp. 115]
Le2703	beta-lytic protease [Lysobacter sp. IB-9374]
Le2716	hypothetical protein [Rudaea cellulosilytica]

Le2717	Hemolysin-type calcium-binding repeat (2 copies) [Candidatus Chloracidobacterium thermophilum B]
Le2718	NA
Le2719	NA
Le2720	NA
Le2726	hypothetical protein [Variovorax sp. CF313, PMI12_00324]
Le2727	transcriptional regulator, AraC family [Variovorax paradoxus EPS]
Le2772	NA
Le2784	hypothetical protein [Rhodanobacter spathiphylli]
Le2785	hypothetical protein [Dyella ginsengisoli]
Le2790	hemolysin-type calcium-binding protein [Clostridium cellulovorans]
Le2830	5-methyltetrahydropteroyltriglutamate--homocysteine methyltransferase [Xanthomonas translucens]
Le2859	NA
Le2871	endoglucanase [Lysobacter sp. IB-9374]
Le2884	conserved hypothetical protein [Variovorax paradoxus]
Le2926	lysine exporter protein [Azoarcus sp. KH32C]
Le2927	NA
Le2933	hypothetical protein [Stenotrophomonas maltophilia Ab55555, A1OC_03568]
Le2955	NA
Le2980	TetR family transcriptional regulator [Myxococcus stipitatus]
Le2997	NA
Le3002	hypothetical protein [Xanthomonas translucens]
Le3019	predicted phosphohydrolase [Hahella chejuensis]
Le3022	hypothetical protein [Xanthomonas translucens]
Le3026	hypothetical protein [Dyella japonica]
Le3046	hypothetical protein [Stenotrophomonas maltophilia Ab55555, A1OC_03423]
Le3060	cytochrome c, class I [Cupriavidus sp. HMR-1]
Le3062	(2Fe-2S)-binding protein [Ralstonia sp. AU12-08]
Le3075	Cupin 2 conserved barrel domain protein [Pseudomonas fulva]
Le3100	Rieske (2Fe-2S) domain-containing protein [Xanthomonas translucens]
Le3180	NA
Le3194	membrane protein [Achromobacter xylosoxidans]
Le3199	NA
Le3201	lysine transporter LysE [Mesorhizobium amorphae]
Le3202	hypothetical protein [Roseomonas sp. B5]
Le3205	hypothetical protein [Chryseobacterium taeanense]
Le3211	hypothetical protein [Uliginosibacterium gangwonense]
Le3226	lipolytic protein G-D-S-L [Collimonas fungivorans Ter331]
Le3232	2-methylcitrate dehydratase [Rhodanobacter sp.]
Le3286	MATE efflux family protein [Thauera sp. 63]
Le3290	bleomycin resistance protein [Rhizobium sp. 42MFCr.1]
Le3292	hypothetical protein [Dyella ginsengisoli]

Le3299	putative methyltransferase [Xanthomonas campestris]
Le3300	hypothetical protein [Xanthomonas sp. SHU199]
Le3307	inner membrane protein [Xanthomonas translucens]
Le3311	hypothetical protein [Variovorax paradoxus EPS, Varpa_1846]
Le3316	hypothetical protein [Xanthomonas arboricola]
Le3322	TfoX domain-containing protein [Collimonas fungivorans]
Le3330	glyoxalase [Stenotrophomonas maltophilia]
Le3333	hypothetical protein [Xanthomonas translucens]
Le3362	hypothetical protein [Nodosilinea nodulosa]
Le3387	NA
Le3393	hypothetical protein [Variovorax sp.]
Le3408	putative transmembrane protein [Stenotrophomonas maltophilia]
Le3429	NAD(P)H dehydrogenase [Xanthomonas sp. SHU199]
Le3452	putative secreted protein [Stenotrophomonas maltophilia D457]
Le3466	lactoylglutathione lyase [Dyella japonica]
Le3473	phospholipase C, phosphocholine-specific [Pseudomonas aeruginosa]
Le3474	hypothetical protein [Klebsiella oxytoca]
Le3475	hypothetical protein [Proteus penneri]
Le3476	protein kinase/helix-hairpin-helix DNA-binding domain-containing protein [Salinisphaera shabanensis]
Le3480	conserved hypothetical protein [Xanthomonas campestris]
Le3483	hypothetical protein [Xanthomonas axonopodis]
Le3491	NA
Le3508	NA
Le3518	hypothetical protein [Acetobacteraceae bacterium]
Le3534	protein of unknown function DUF336 [Candidatus Solibacter usitatus Ellin6076]
Le3541	cytochrome C551 [Pseudoxanthomonas sp. GW2]
Le3542	hypothetical protein [Xanthomonas sacchari]
Le3554	periplasmic glucan biosynthesis protein, MdoG [Roseomonas cervicalis ATCC 49957]
Le3555	Glucans biosynthesis glucosyltransferase H [Xanthomonas campestris]
Le3595	SMP-30/gluconolaconase/LRE-like region-containing protein [Pseudoxanthomonas suwonensis]
Le3616	NA
Le3668	NA
Le3689	hypothetical protein [Gemmata obscuriglobus]
Le3691	hypothetical protein [Pseudomonas mendocina]
Le3704	hypothetical protein [Xanthomonas axonopodis]
Le3714	hypothetical protein [Dyella japonica]
Le3732	NA
Le3757	hypothetical protein [Xanthomonas sp. SHU199]
Le3760	membrane protein [Xanthomonas campestris]
Le3811	hemin storage protein [Stenotrophomonas maltophilia]
Le3812	alpha-1, 2-mannosidase [Rhodanobacter thiooxydans LCS2]
Le3814	NA

Le3815	NA
Le3847	hypothetical protein [Xanthomonas sacchari]
Le3858	putative phospholipase c precursor protein [Xanthomonas albilineans]
Le3884	NA
Le3904	hypothetical protein [Janthinobacterium sp. CG3]
Le3905	MFS transporter [Serratia marcescens]
Le3915	hypothetical protein [Rubrivivax gelatinosus]
Le3917	endonuclease [Stenotrophomonas maltophilia]
Le3925	NA
Le3940	NA
Le3944	hypothetical protein [Candidatus Solibacter]
Le3991	hypothetical protein [Pseudoxanthomonas spadix]
Le4025	NA
Le4030	NA
Le4067	membrane protein [Pseudomonas chlororaphis]
Le4071	putative transmembrane protein [Stenotrophomonas maltophilia]
Le4074	lysine exporter protein LysE/YggA [Pseudomonas sp. CF149]
Le4102	hypothetical protein [Xanthomonas sp. SHU199]
Le4115	NA
Le4136	conserved hypothetical protein [Xanthomonas citri]
Le4138	hypothetical protein [Xanthomonas fragariae]
Le4141	putative transcriptional regulator [Mesorhizobium metallidurans]
Le4163	NA
Le4180	hypothetical protein [Xanthomonas sacchari]
Le4187	NA
Le4199	putative membrane protein [Mesorhizobium amorphae]
Le4224	hypothetical protein [Bifidobacterium adolescentis]
Le4225	hypothetical protein BIFADO_01105, partial [Bifidobacterium adolescentis L2-32]
Le4231	NA
Le4252	hypothetical protein [Methylobacterium radiotolerans]
Le4258	hypothetical protein [Stenotrophomonas maltophilia]
Le4260	sugar ABC transporter substrate-binding protein [Xanthomonas translucens]
Le4261	binding-protein-dependent transport systems inner membrane component
Le4262	sugar ABC transporter permease [Stenotrophomonas maltophilia]
Le4263	hypothetical protein [Xanthomonas translucens]
Le4272	NA
Le4275	MFS transporter [Dyella ginsengisoli]
Le4301	NA
Le4302	NA
Le4306	NAD(P)H dehydrogenase [Pseudomonas aeruginosa]
Le4323	Glyoxalase/bleomycin resistance protein/dioxygenase [Pseudoxanthomonas suwonensis]
Le4332	hypothetical protein [Stenotrophomonas maltophilia]
Le4337	NA

Le4383	hypothetical protein [Sphingobium sp.]
Le4386	membrane protein [Sphingobium lactosutens DS20]
Le4387	membrane protein [Stenotrophomonas maltophilia]
Le4388	hypothetical protein [alpha proteobacterium LLX12A]
Le4421	hypothetical protein [Xanthomonas oryzae]
Le4422	copper resistance protein M [Xanthomonas alfalfae subsp. citrumelonis]
Le4427	NA
Le4432	FAD-binding 9 siderophore-interacting domain-containing protein
Le4467	cycloisomaltooligosaccharide glucanotransferase [Xanthomonas sp. SHU199]
Le4474	kanamycin nucleotidyltransferase, partial [Catenibacterium mitsuokai DSM 15897]
Le4527	putative uncharacterized protein [Burkholderia sp. RPE64]
Le4557	chemotaxis protein CheW [Pseudomonas fluorescens]
Le4558	putative chemotaxis-related methyltransferase [Pseudomonas fluorescens Pf0-1]
Le4559	chemotaxis protein CheW [Herbaspirillum frisingense]
Le4572	lanthionine synthetase C-like protein [Lysobacter sp. ATCC 53042]
Le4573	hypothetical protein [Lysobacter sp. ATCC 53042]
Le4588	beta-lactamase domain-containing protein [Lysobacter sp. ATCC 53042]
Le4595	putative Mg(2+) transporter [Lysobacter sp. ATCC 53042]
Le4598	major facilitator family transporter [Lysobacter sp. ATCC 53042]
Le4604	cytochrome-c peroxidase [Lysobacter sp. ATCC 53042]
Le4640	hydrolase [Burkholderia pyrrocinia]
Le4641	hypothetical protein [Rudaea cellulosilytica]
Le4644	hypothetical protein [Xanthomonas sp. SHU199]
Le4645	hypothetical protein [Rhodanobacter fulvus]
Le4648	transcriptional regulator [Fluoribacter dumoffii]
Le4650	hypothetical protein [uncultured bacterium, ACD_54C00931G0002]
Le4651	hypothetical protein [Xanthomonas sp. SHU199]
Le4683	NA
Le4696	NA
Le4697	NA
Le4734	beta-mannosidase [Stenotrophomonas maltophilia]
Le4738	alpha-1 2-mannosidase [Xanthomonas axonopodis]
Le4742	copper homeostasis protein CutC [Stenotrophomonas maltophilia]
Le4752	NA
Le4755	NA
Le4758	hypothetical protein [Lamprocystis purpurea]
Le4759	glycosyl transferase [Vibrio breoganii]
Le4791	NA
Le4792	NA
Le4796	NA
Le4798	putative exported protein [Xanthomonas translucens]
Le4800	hypothetical protein [Achromobacter xylosoxidans]
Le4801	hypothetical protein [Acidovorax sp. MR-S7, AVS7_01837]

Le4802	hypothetical protein [Uliginosibacterium gangwonense]
Le4803	NA
Le4804	NAD dependent epimerase/dehydratase [Pseudomonas sp. GM80]
Le4805	putative thiol-disulfide oxidoreductase DCC [Burkholderia gladioli]
Le4816	NA
Le4832	hypothetical protein [Rudaea cellulosilytica]
Le4857	hypothetical protein [Methylomonas methanica]
Le4860	hypothetical protein [Stenotrophomonas maltophilia Ab55555, A1OC_03207]
Le4876	hypothetical protein [Xanthomonas vesicatoria]
Le4888	alkane 1-monooxygenase [Xanthomonas sacchari]
Le4891	NA
Le4909	NA
Le4911	NA
Le4916	NA
Le4918	hypothetical protein [Cellvibrio sp.]
Le4933	hypothetical protein [Halomonas anticariensis]
Le4945	hypothetical protein [Xanthomonas sacchari]
Le4951	hypothetical protein [Rhodanobacter sp.]
Le4968	glyoxalase [Kaistia granuli]
Le4977	hypothetical protein [Variovorax sp.]
Le4978	hypothetical protein [Acetobacter tropicalis]
Le4980	carbohydrate-binding CenC domain protein [Conexibacter woesei]
Le4981	NA
Le4983	alpha/beta hydrolase fold protein [Pseudoxanthomonas suwonensis]
Le5000	protein of unknown function DUF1470 [Granulicella tundricola MP5ACTX9]
Le5013	NA
Le5022	hypothetical protein [Xanthomonas translucens]
Le5025	hypothetical protein [Enterococcus moraviensis]
Le5040	NA
Le5047	putative membrane protein [Xanthomonas translucens]
Le5053	hypothetical protein [Xanthomonas sp. SHU199]
Le5079	NA
Le5104	putative transcriptional regulator, MarR family
Le5141	NA
Le5159	NA
Le5160	NA
Le5161	NA
Le5167	NA
Le5172	NA
Le5173	NA
Le5202	hypothetical protein, partial [Xanthomonas perforans]
Le5236	NA
Le5260	NA

Table S2. Distribution of Le4759 and Le0100 in non-*Lysobacter* bacterial genomes

Organisms	LuxR	LuxI	Le4759	Le0100
Luteimonas aquatica RIB1-20	WP_242108327.1	none	WP_242113092.1	none
Arenimonas composti TR7-09	none	none	WP_026816606.1	none
Thermomonas alba SYSU G04536	none	none	WP_240125151.1	none
Halomonas aerodenitrificans MCCC 1A11058	none	none	WP_234255712.1	none
Tahibacter caeni BUT-6	WP_257385461.1	none	none	WP_257386727.1
Pseudomonas fluorescence 2P24	AAT42219.1	AAT42217.1	none	none
Pectobacterium carotovorum PccS1	WP_181848026.1	WP_015842234.1	none	none
Pseudomarimonas arenosa CAU 1598	WP_225444625.1	WP_192030098.1	none	none
Aquimonas voraii DSM 16957	WP_176764180.1	WP_176764181.1	WP_091242174.1	none
Nitrosospira lacus APG3	WP_004180566.1	WP_004180210.1	WP_004180885.1	none
Nitrosospira briensis C-128	WP_025041731.1	WP_025041701.1	WP_025040903.1	none
Candidatus Nitrotoga AM1P	WP_173052046.1	WP_242007018.1	WP_173052373.1	none
Nitrosovibrio tenuis Nv1	WP_090826092.1	WP_090826837.1	WP_090827938.1	none
Nitrosospira multiformis Nl1	WP_074630300.1	WP_218124298.1	WP_074630447.1	none
Ahniella affigens D13	WP_106891580.1	WP_106891581.1	WP_170113451.1	none
Paraburkholderia dipogonis ICMP 19430	WP_106300542.1	WP_134460123.1	WP_134457033.1	none

Table S3. Strains and plasmids used in this study

Strains and plasmids	Characteristics[a]	Source
**Wild-type strains**		
Lysobacter enzymogenes OH11	Wild type, Km^{R}	(1)
Pseudomonas fluorescence 2P24	Wild type	(2)
Pectobacterium carotovorum PccS1	Wild type,Rif^{R}	
JZA1	*Agrobacterium tumefaciens* carrying an AHL reporter plasmid with a traG::lacZ fusion, Sp^{R}， Gm^{R}, Tc^{R}	(3)
Escherichia coli		
DH5α	Host strain for molecular cloning	(4)
XL1-Blue MRF' Kan	Host for bacterial two-hybrid assay	(4)
BL21(DE3)	Host strain for protein expression, Km^{R}	(4)
JMP	JM109 with a P_{BAD}:: *pcoI* fragment insertion on chromosome	Laboratory stock
**In-frame deletion mutants**		
Δ*pcoI*	In-frame deletion of *pcoI* in strain 2P24, Km^{R}	(2)
**Overexpression strains**		
2P24 (*gfp*)	2P24 harbouring plasmid pBBR-*gfp*, Gm^{R}	This study
2P24 (*Le4759*)	2P24 harbouring plasmid pBBR-*Le4759*, Gm^{R}	This study
JMP((*gfp*)	JMP harbouring plasmid pBBR-*gfp*, Gm^{R}	This study
JMP(*Le4759*)	JMP harbouring plasmid pBBR-*Le4759*, Gm^{R}	This study
JMP(EV)	JMP harbouring pBBR empty plasmid, Gm^{R}	This study
JMP(*momL*)	JMP harbouring plasmid pBBR-*momL*, Gm^{R}	This study
PccS1(*Le4759*)	PccS1 harbouring plasmid pBBR-*Le4759*, Gm^{R},Rif^{R}	This study
PccS1 (*gfp*)	PccS1 harbouring plasmid pBBR-*gfp*, Gm^{R},Rif^{R}	This study
PccS1(*Le4759*^{R70L})	PccS1 harbouring plasmid pBBR-*Le4759*^{R70L}, Gm^{R},Rif^{R}	This study
PccS1(*Le4759*^{R84H})	PccS1 harbouring plasmid pBBR-*Le4759*^{R84H}, Gm^{R},Rif^{R}	This study
PccS1(*Le4759*$^{1-200}$)	PccS1 harbouring plasmid pBBR-*Le4759*$^{1-200}$, Gm^{R},Rif^{R}	This study
PccS1(*Le4759*$^{212-383}$)	PccS1 harbouring plasmid pBBR-*Le4759*$^{212-383}$, Gm^{R},Rif^{R}	This study
Δ*pcoI* (*gfp*)	Δ*pcoI* harbouring plasmid pBBR-*gfp*, Gm^{R}	This study
Δ*pcoI* (*Le4759*)	Δ*pcoI* harbouring plasmid pBBR-*Le4759*, Gm^{R}	This study
Δ*pcoI* (*momL*)	Δ*pcoI* harbouring plasmid pBBR-*momL*, Gm^{R}	This study
DH5α (*carI & Le4759*)	DH5α harbouring plasmid pBBR-*Le4759* and pUCP26-*carI* Tc^{R}, Gm^{R}	This study
DH5α (*carI & gfp*)	DH5α harbouring plasmid pBBR-*gfp* and pUCP26-*carI* Tc^{R}, Gm^{R}	This study
DH5α (*carI &* EV)	DH5α harbouring pBBR empty plasmid and pUCP26-*carI* Tc^{R}, Gm^{R}	This study
DH5α (*lbsI & Le4759*)	DH5α harbouring plasmid pBBR-*Le4759* and pUCP26-*lbsI* Tc^{R}, Gm^{R}	This study
DH5α (*lbsI & gfp*)	DH5α harbouring plasmid pBBR-*gfp* and pUCP26-*lbsI* Tc^{R}, Gm^{R}	This study
DH5α (*lbsI &* EV)	DH5α harbouring pBBR empty plasmid and pUCP26-*lbsI* Tc^{R}, Gm^{R}	This study
**Plasmids**		This study
pBBR-MCS5	Broad-host-vector with a P_{lac} promoter, Gm^{R}	(5)
pTRG	The plasmid used for protein expression in bacterial two-hybridization assay, Tc^{R}	(6)
pBT	The plasmid used for protein expression in bacterial two-hybridization assay, Chlo^{R}	(6)
pBT-*Le4759*	pBT with the coding region of *Le4759* from strain OH11, Chl^{R}	This study
pBT-*gacS*	pBT containing the coding region of *gacS*	(4)
pTRG-*pcoI*	pTRG with the coding region of *pcoI,* Tc^{R}	This study
pTRG-*lbsI*	pTRG with the coding region of *lbsI,* Tc^{R}	This study
PTRG-*carI*	pTRG containing the coding region of *carI,* Tc^{R}	This study
pTRG-*gacS*	pTRG containing the coding region of *gacS,* Tc^{R}	This study
pBBR-*gfp*	pBBR-MCS5 with the coding region of *gfp*, Gm^{R}	This study
pBBR-*pcoI*	pBBR-MCS5 with the coding region of *pcoI*, Gm^{R}	This study
pBBR-*Le4759*	pBBR-MCS5 with the coding region of *Le4759*, Gm^{R}	This study
pBBR-*Le4759*^{R70L}	pBBR-MCS5 with the coding region of *Le4759*^{R70L}, Gm^{R}	This study
pBBR-*Le4759*^{R84H}	pBBR-MCS5 with the coding region of *Le4759*^{R84H}, Gm^{R}	This study
pBBR-*Le4759*$^{1-200}$	pBBR-MCS5 with the coding region of *Le4759*$^{1-200}$, Gm^{R}	This study
pBBR-*Le4759*$^{212-383}$	pBBR-MCS5 with the coding region of *Le4759*$^{212-383}$, Gm^{R}	This study
pBBR-*momL*	pBBR-MCS5 with the coding region of *momL*, Gm^{R}	This study
pUCP26-*pcoI*	pUCP26 with the coding region of *pcoI*, Gm^{R}	This study
pUCP26-*carI*	pUCP26 with the coding region of *carI*, Gm^{R}	This study
pUCP26-*lbsI*	pUCP26 with the coding region of *lbsI*, Gm^{R}	This study

[a]Km^{R}, Gm^{R} Amp^{R}, Tc^{R}, Chlo^{R}, Sp^{R}, kanamycin, gentamicin, ampicillin, tetracycline, chloramphenicol, spectinomycin resistance, respectively.

Table S4. Primers used in this study

Primer	Sequence (5'-3')[a]	Purpose
**Overexpression**		
Le4759-F	AGGGAACAAAAGCTGGGTACCgATGCGCATCTTGTTCCTGACCG (*Kpn*I)	To amplify a 1257-bp fragment of *Le4759* fused with a FLAG tag
Le4759-FLAG-R	CGCGGTGGCGGCCGCTCTAGAttACTTATCGTCGTCATCCTTGTAATCCT CCGGCGTGCGCAACAGGC (*Xba*II)	
momL-F	CCCAAGCTTgAtgaatatgaaaaagctacttgtac (*Hin*dIII)	To amplify a 1377-bp fragment of *momL*
momL-R	TGCTCTAGAttattgtaaatagttgggtgcctgg(*Xba*II)	
Le4759$^{212-383}$-F	CGCGGTGGCGGCCGCTCTAGATTACTTATCGTCGTCATCCTTGTAATCGC CGTTGAGCACGACGTGGATCTT(*Kpn*I)	To amplify a 516-bp downstream homologue of *Le4759*$^{212-383}$
Le4759$^{1-200}$-FLAG-R	CGCGGTGGCGGCCGCTCTAGATCACTTATCGTCGTCATCCTTGTAATCC ACGCTGGCGGCGAGCACGGCGAAG (*Xba*II)	To amplify a 600-bp upstream homologue of *Le4759*$^{1-200}$ fused with a FLAG tag
Le4759^{R70L}-F	TCCGGGTGGTGCtGGTCAAGACC	To amplify a 1061-bp downstream homologue of *Le4759*^{R70L}
Le4759^{R70L}-R	GGTCTTGACCAGCACCACCCGGA	To amplify a 219-bp upstream homologue of *Le4759*^{R70L}
Le4759^{R84H}-F	GCTTCCTCAAGCaCACCCTGGAC	To amplify a 1019-bp downstream homologue of *Le4759*^{R84H}
Le4759^{R84H}-R	GTCCAGGGTGTGCTTGAGGAAGC	To amplify a 216-bp upstream homologue of *Le4759*^{R84H}
gfp-FLAG-F	CCCAAGCTTgATGAGTAAAGGTGAAGAACTGT (*Hin*dIII)	To amplify a 717-bp fragment upstream of *gfp* fused with a FLAG tag
gfp-FLAG-R	TGCTCTAGATCACTTATCGTCGTCATCCTTGTAATCTTTGTAGAGTTCAT CCATG (*Xba*I)	
**Protein expression and Pull-down assays**		
pET30a-*pcoI*-F	GGAATTCCATATGAAATACTTAATAGACAAACGCGAG(*Nde*I)	To amplify a 576-bp fragment of *pcoI*
pET30a-*pcoI*-R	CCCAAGCTTACCACCGTTGACGGCGTCCAACG (*Hin*dIII)	
pET30a-*carI*-F	ACTTTAAGAAGGAGATATACATATGATGTTAGAGATATTTGATG(*Nde*I)	To amplify a 651-bp fragment of *carI*
pET30a-*carI*-R	GGTGCTCGAGTGCGGCCGCAAGCTTAGCCTGTACAATAGCGGCAG (*Hin*dIII)	
pET30a-*lbsI*-F	ACTTTAAGAAGGAGATATACATATGATGACCCGTATTGCCATCGGC(*Nde*I)	To amplify a 618-bp fragment of *lbsI*
pET30a-*lbsI*-R	GGTGCTCGAGTGCGGCCGCAAGCTTGGCTGCGCGGGCGATGTCGAGT (*Hin*dIII)	
pET30a-*Le4759*-F	ACTTTAAGAAGGAGATATACATATGATGCGCATCTTGTTCCTGACCG(*Nde*I)	To amplify a 1257-bp fragment of *Le4759*
pET30a-*Le4759*-R	GGTGCTCGAGTGCGGCCGCAAGCTTCTCCGGCGTGCGCAACAGGC (*Hin*dIII)	
**Co-IP assays**		
Le4759-F	AGGGAACAAAAGCTGGGTACCgATGCGCATCTTGTTCCTGACCG (*Kpn*I)	To amplify a 1257-bp fragment of *Le4759* fused with a FLAG tag
Le4759-FLAG-R	CGCGGTGGCGGCCGCTCTAGAttACTTATCGTCGTCATCCTTGTAATCCT CCGGCGTGCGCAACAGGC (*Xba*II)	
pcoI-His-F	CTATGACCATGATTACGAATTCgATGAAATACTTAATAGACAAACGCG (*Eco*RI)	To amplify a 576-bp fragment of *pcoI* fused with a His tag
pcoI-His-R	GCAGGTCGACTCTAGAGGATCCTTAATGATGATGATGATGGTGACCACC GTTGACGGCGTCCAACG (*Bam*HI)	
carI-His-F	CTATGACCATGATTACGAATTCgATGTTAGAGATATTTGATG	To amplify a 651-bp fragment of *CarI* fused with a His tag
carI-His-R	GCAGGTCGACTCTAGAGGATCCTTAATGATGATGATGATGGTGAGCCTG TACAATAGCGGCAG	
lbsI-His-F	CTATGACCATGATTACGAATTCgATGACCCGTATTGCCATCGGC	To amplify a 618-bp fragment of *LbsI* fused with a His tag
lbsI-His-R	CAGGTCGACTCTAGAGGATCCTCAATGATGATGATGATGGTGGGCTGCG CGGGCGATGTCGAGT	
gfp-FLAG-F	CCCAAGCTTgATGAGTAAAGGTGAAGAACTGT (*Hin*dIII)	To amplify a 717-bp fragment of *gfp* fused with a FLAG tag
gfp-FLAG-R	TGCTCTAGATCACTTATCGTCGTCATCCTTGTAATCTTTGTAGAGTTCAT CCATG (*Xba*I)	
**Bacterial two-hybrid assays**		
pBT-*Le4759*-F	TGGCGCGGCCGCATCGAATTCCATGCGCATCTTGTTCCTGAC (*Eco*RI)	To amplify a 1257-bp fragment of *Le4759* and cloned into pBT
pBT-*Le4759*-R	AATTAATTAACTCGAGGATCCTTACTCCGGCGTGCGCAACAGG (*Bam*HI)	

pTRG-*pcoI*-F	AAACCAGAGGCGGCCGGATCCGATGAAATACTTAATA (*Bam*HI)	To amplify a 576-bp fragment of *pcoI* and cloned into pTRG
pTRG-*pcoI*-R	GCGCCAGCTCAGACTGAATTCTTAACCACCGTTGACGGC (*Eco*RI)	
pTRG-*carI*-F	AAACCAGAGGCGGCCGGATCCATGTTAGAGATATTTGATG (*Bam*HI)	To amplify a 651-bp fragment of *carI* and cloned into pTRG
pTRG-*carI*-R	GCGCCAGCTCAGACTGAATTCTTAAGCCTGTACAATAGCGG (*Eco*RI)	
pTRG-*lbsI*-F	AAACCAGAGGCGGCCGGATCCATGACCCGTATTGCCATCGG (*Bam*HI)	To amplify a 618-bp fragment of *lbsI* and cloned into pTRG
pTRG-*lbsI*-R	GCGCCAGCTCAGACTGAATTCTCAGGCTGCGCGGGCGATGTC (*Eco*RI)	

[a]Restricted digestion enzyme site was underlined.

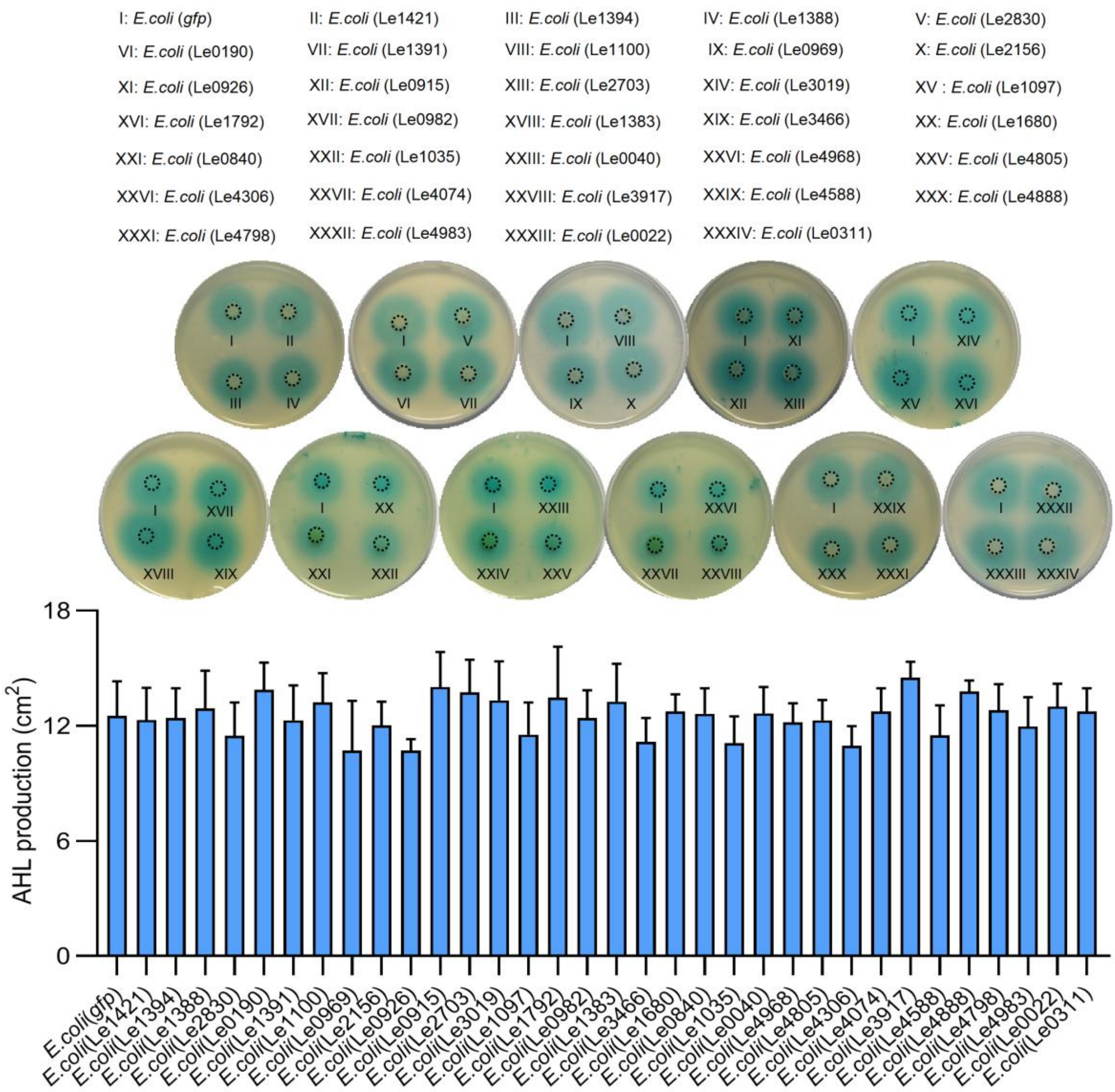

Figure S1. The *Lysobacter* genes was screened to identify targets that block AHL production in *E. coli* JM109 expressing the active AHL synthase gene *pcoI*. The 34 selected *Lysobacter* genes were those only conservatively shared by non-AHL-producing *Lysobacter* but absent in AHL-producing species. Its details are shown in the Table S1 with red highlight. PcoI is an AHL synthase from *Pseudomonas fluorescence* 2P24.

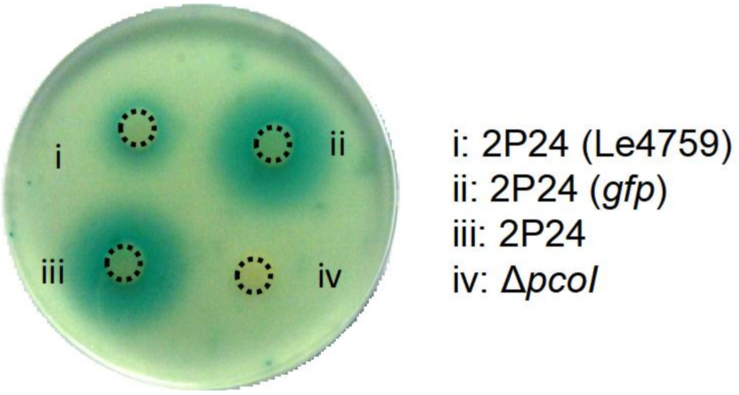

Figure S2. Representative images showing the production of AHL in *Pseudomonas fluorescence* 2P24 expressing Le4759. AHL production was determined by the AHL biosensor JZA1, and the AHL quantification data were shown in Figure 1F.

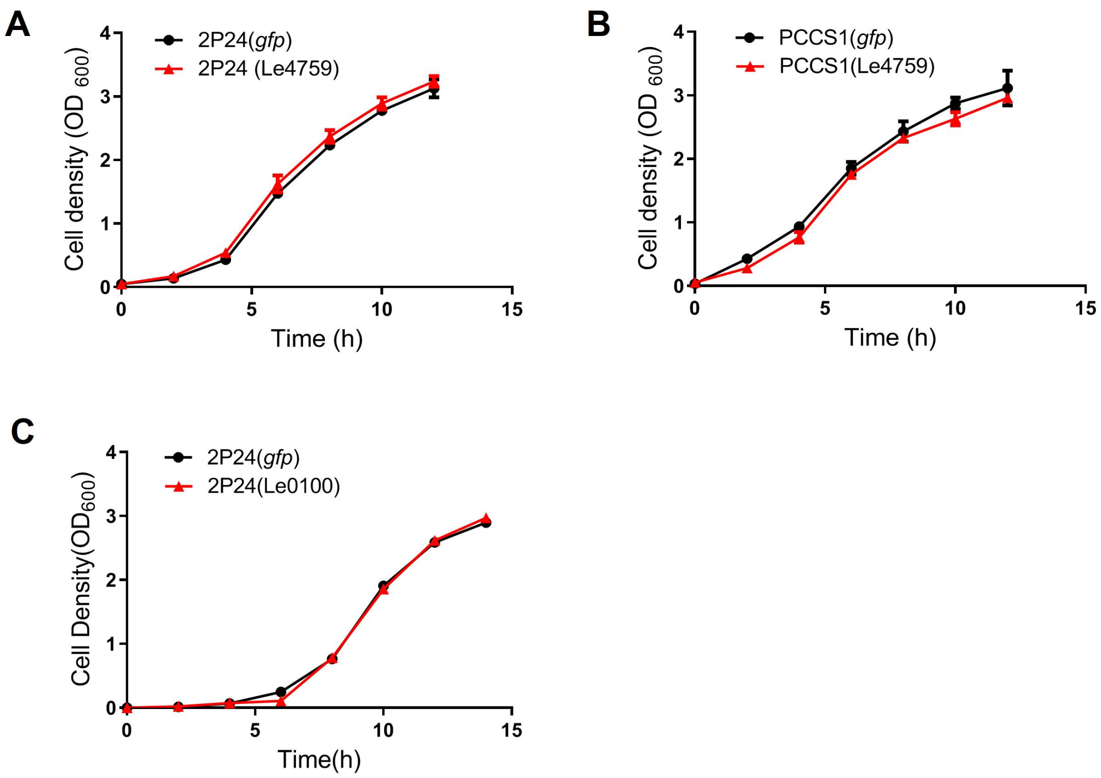

Figure S3. Heterogeneous expression of *Le4759* and *Le0100* in *Pseudomonas fluorescence* 2P24 did not alter the normal growth ability of the bacteria.

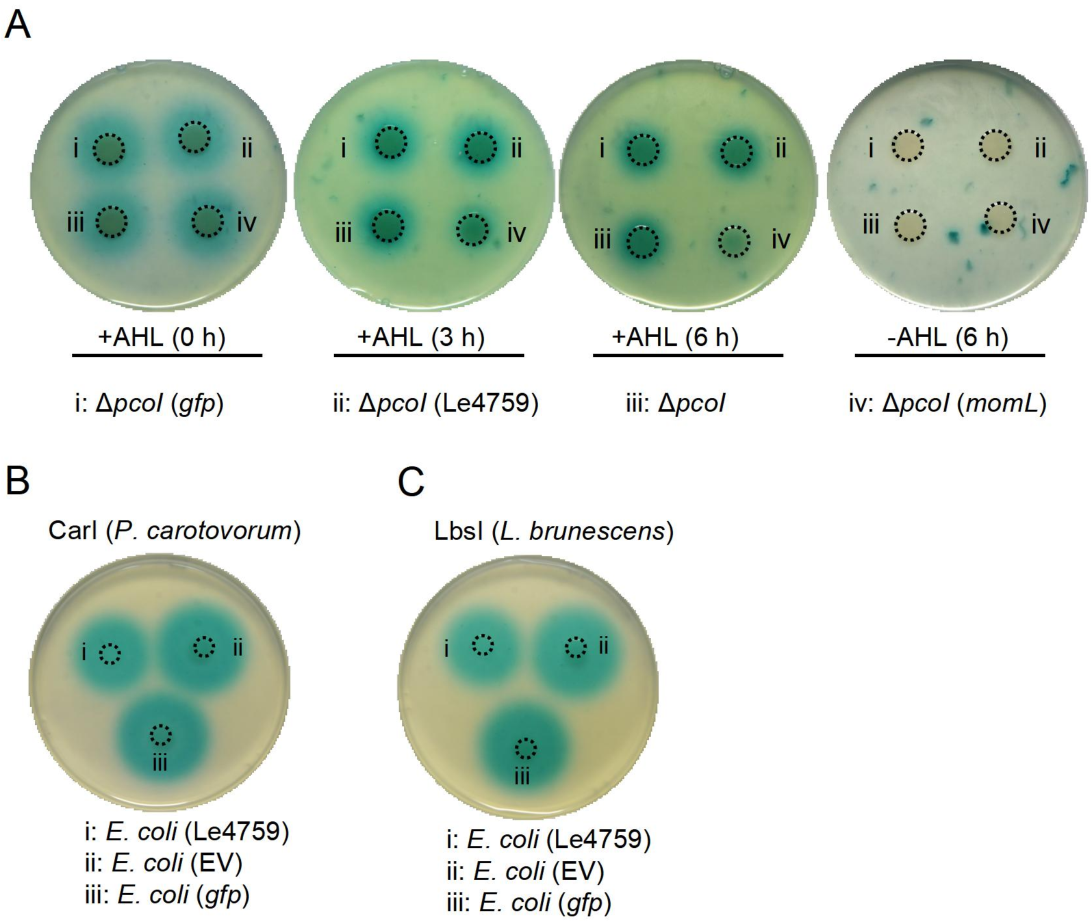

Figure S4. Representative images of AHL production as determined by the AHL biosensor JZA1. (A) Le4759 failed to degrade the commercial AHL standard (3-oxo-C10-HSL)**. (B-C)** Le4759 blocked the CarI- or LbsI-dependent AHL production in *E. coli*. AHL quantification data in panels A, B and C were shown in Figure 2A, 2F and 2G, respectively.

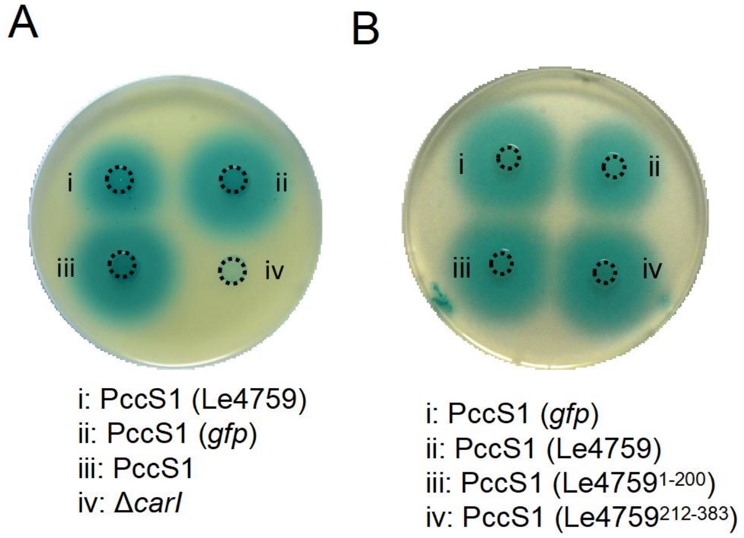

Figure S5. Representative images showing AHL production in *Pectobacterium carotovorum* PccS1 expressing Le4759 and its two truncated variants. AHL production was determined by the AHL biosensor JZA1. AHL quantification data in panels A and B were shown in Figure 3A and 3G, respectively.

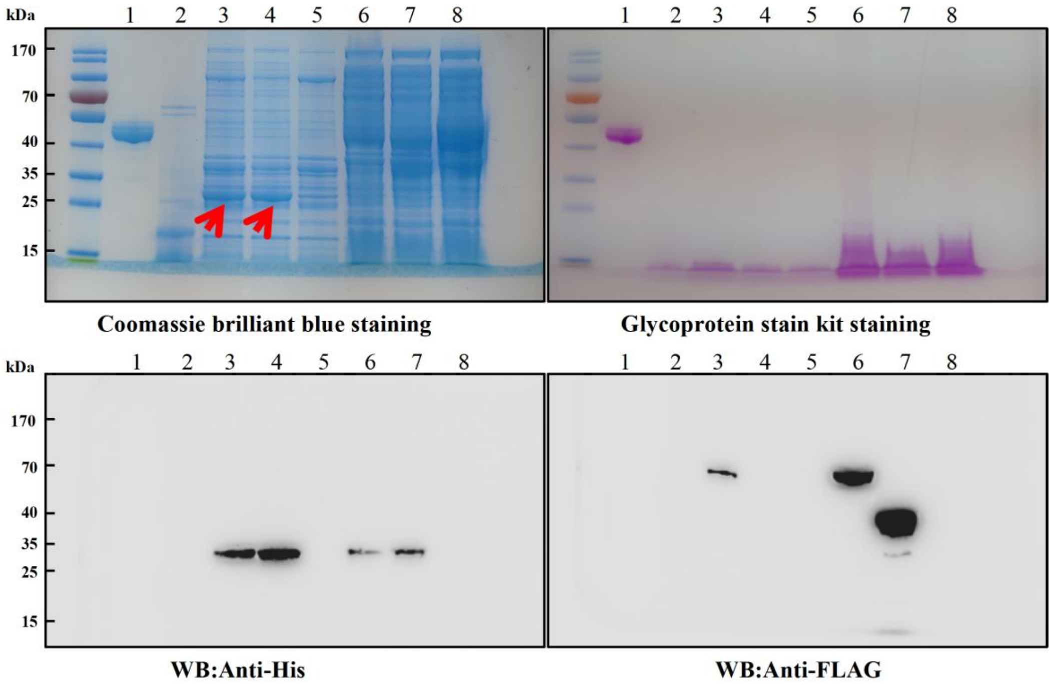

Figure S6. Co-expression of the *Le4759* and *pcoI* gene in *E. coli* did not detect the glycosyltransferase activity of Le4759. We co-transformed the pUCP-PcoI-His and pBBR-Le4759-FLAG or pBBR-GFP-FLAG (negative control) in *E. coli* DH5α, and drove the expression of *pcoI* and Le4759 by a constitutive promoter from the corresponding plasmid.The corresponding strains were collected and broken by ultrasound, and the supernatant was incubated with anti-His magnetic beads to successfully enriched the PcoI-His protein under the condition of expressing Le4759 and GFP. Glycoprotein staining assay revealed that Le4759 did not appear to be an active glycosyltransferase for glycosylated PcoI in *E. coli*. Line1: Positive control, containing 16% glycoprotein; Line2: Negative control, no glycoprotein; Line3-5： PcoI+Le4759, PcoI+GFP, *E.coli* DH5α supernatant enriched with anti-His magnetic beads, respectively; Line6-8 ： PcoI+Le4759, PcoI+GFP, *E. coli* DH5α cell fragmentation supernatant, respectively. The red arrow indicates the position of the PcoI.

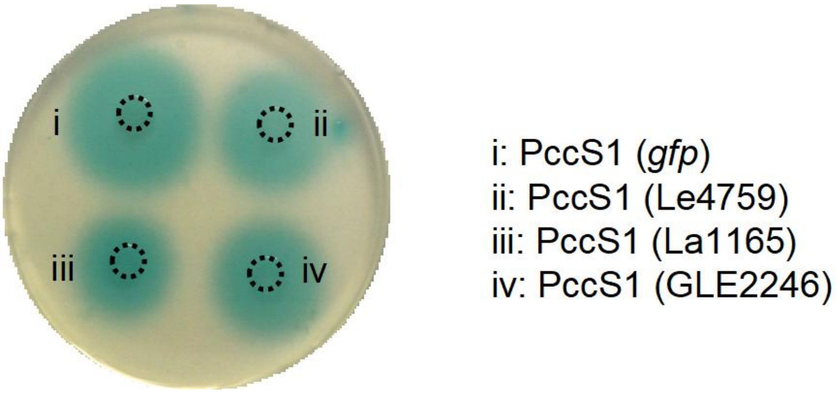

Figure S7. Representative images showing the production of AHL in *Pectobacterium carotovorum* PccS1 expressing Le4759 and its two homologous genes (La1165 and GLE2246). AHL production was determined by the AHL biosensor JZA1, and AHL quantification data were shown in Figure 3H.

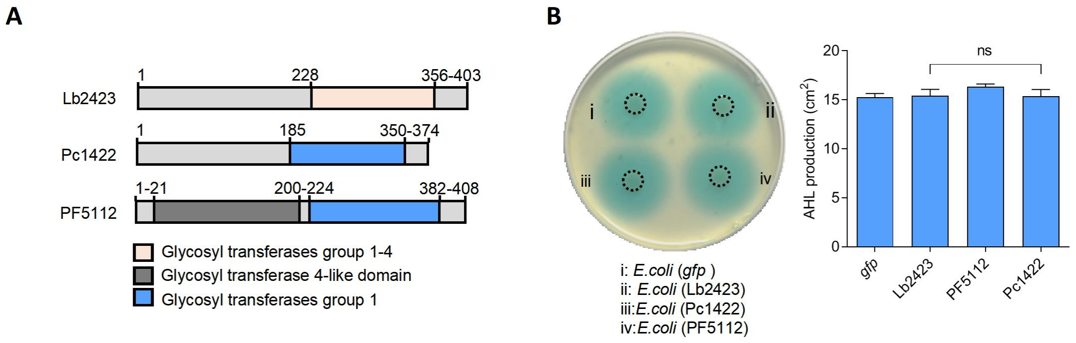

Figure S8. Detection of AHL-quenching activity of three Le4759-unrelated, glycosyltransferase-domain containing genes. (A) Prediction domains of three Le4759-unrelated, glycosyltransferase-domain containing genes (*Lb2423*, *Pc1422* and *PF5112*) of *Lysobacter brunescens* OH21, *Pectobacterium carotovorum* PccS1 and *Pseudomonas fluorescence* 2P24. **(B)** Ectopic expression of the *Lb2423*, *Pc1422* and *PF5112* gene in recombinant, *pcoI*-expressing *E. coli* had no detectable AHL-quenching activity. Recombinant *E. coli* was induced to produce AHL by adding 0.1% arabinose. NCBI reference sequence accession numbers: WP_194431416.1 (Pc1422), WP_134925924.1 (PF5112).

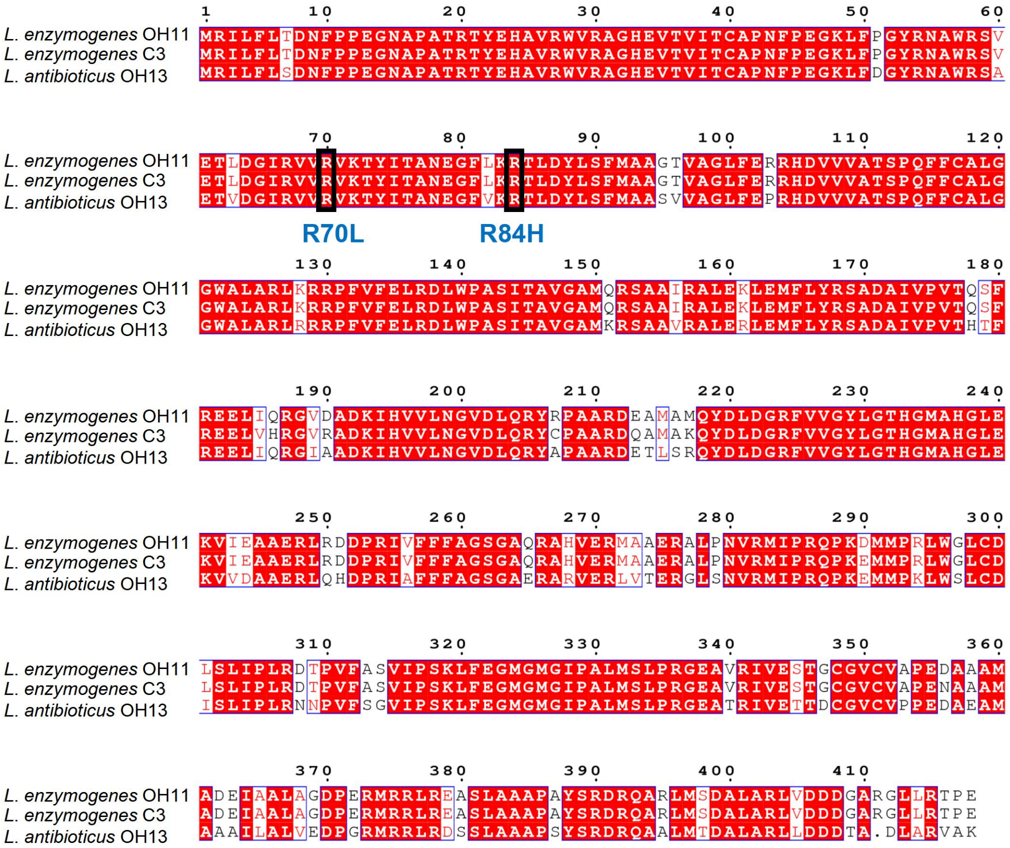

Figure S9. Sequence alignment of Le4759 and its two homologues (GLE2246 and La1165). Boxes indicates the positions of conserved amino residues R70 and R84. GLE2246 and La1165 are homologues of Le4759 from the non-AHL-producing *L. enzymogenes* C3 and *L. antibioticus* OH13, respectively.

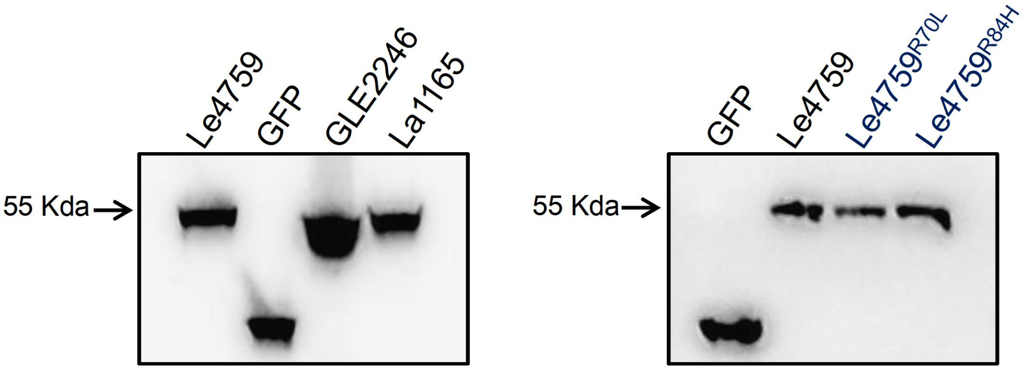

Figure S10. Western blotting analysis of protein expression in *Pectobacterium carotovorum* PccS1. The left panel represents the heterogeneous expression of Le4759 and its two homologous genes (*GLE2246* and *La1165*) in *P. carotovorum* PccS1. The right panel represents the expression of two Le4759 point mutant variants (Le4759^{R70L} and Le4759^{R84H}) in *P. carotovorum* PccS1.

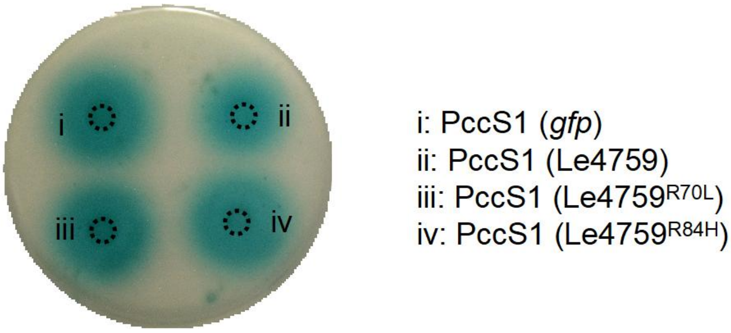

Figure S11. Representative images showing the production of AHL in *Pectobacterium carotovorum* PccS1 expressing Le4759 and its two point mutant variants. AHL production was determined by the AHL biosensor JZA1, and the AHL quantification data were shown in Figure 3I.

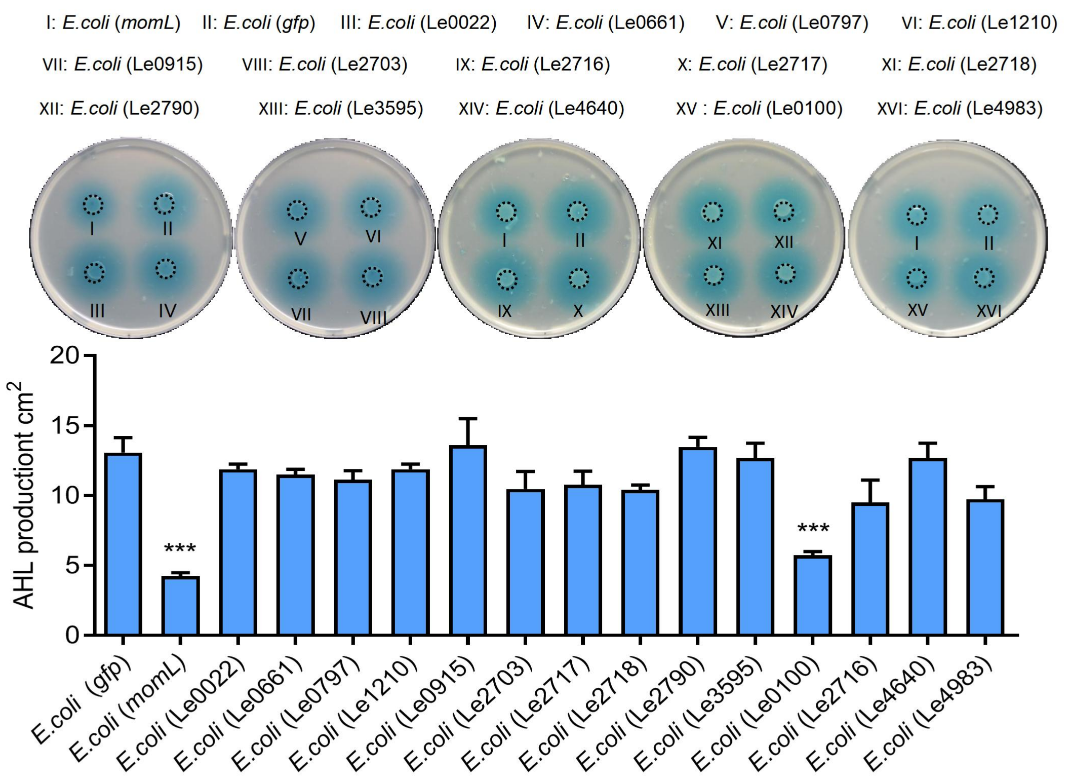

Figure S12. Additional 14 *Lysobacter* genes were screened to identify targets that could prevent AHL production in *E. coli* JM109 expressing the active AHL synthase gene PcoI. The 14 *Lysobacter* genes selected were those that were conservatively shared only by non-AHL-producing *Lysobacter* but not present in AHL-producing species. Its details were shown in a supplemental excel table with blue highlight. PcoI is an AHL synthase from *Pseudomonas fluorescence* 2P24.

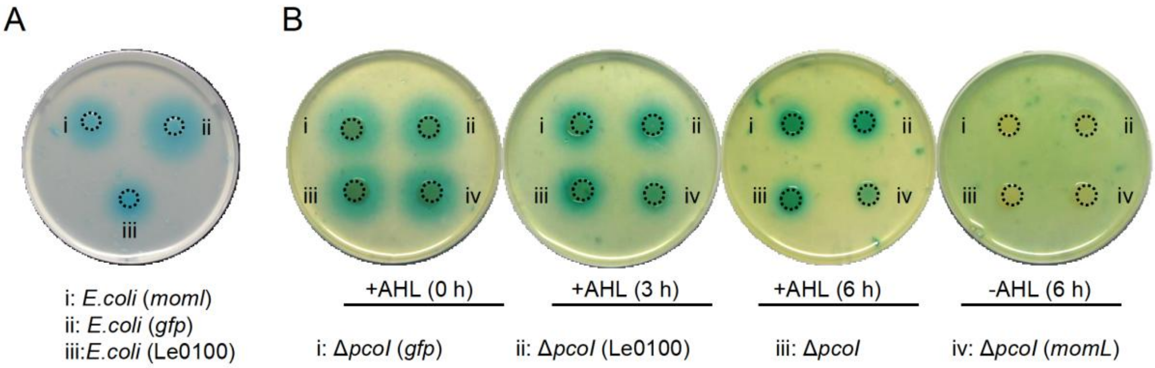

Figure S13. Representative images of AHL production as determined by the AHL biosensor JZA1. (A) Le0100 blocked the PcoI-dependent AHL production in *E. coli*. **(B)** Le0100 failed to degrade the commercial AHL standard (3-oxo-C10-HSL)**.** AHL quantification data in panels A and B were shown in Figure 4A and 4B, respectively.

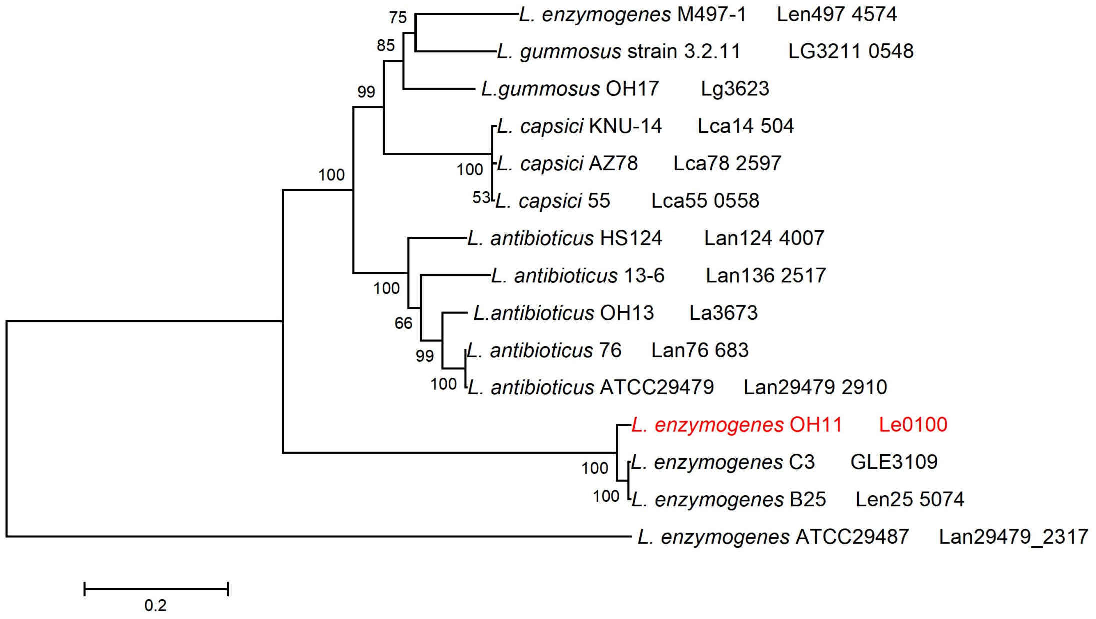

Figure S14. **Phylogenetic tree of Le0100 and its homologues from 15 selected non-AHL-producing *Lysobacter* species.**

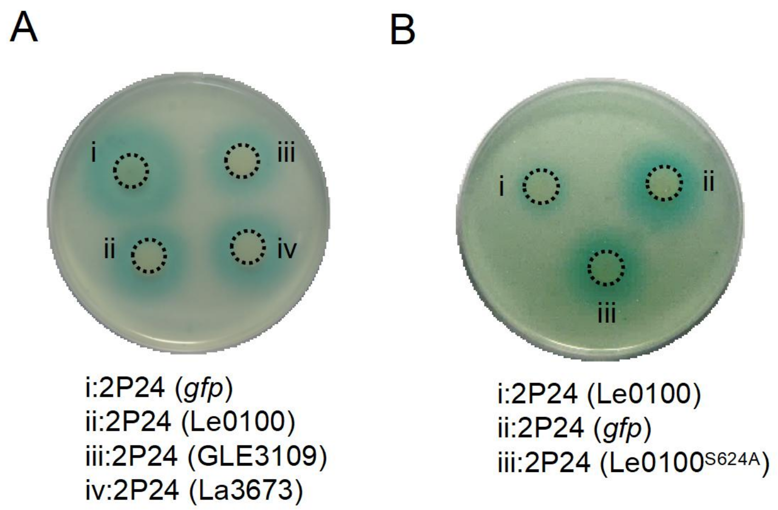

Figure S15. Representative images showing AHL production of *Pseudomonas fluorescence* 2P24 expressing Le0100 and its two homologous genes (A) as well as its point mutant variant (B). AHL quantification data in panels A and B were shown in Figure 4C and 4D, respectively.

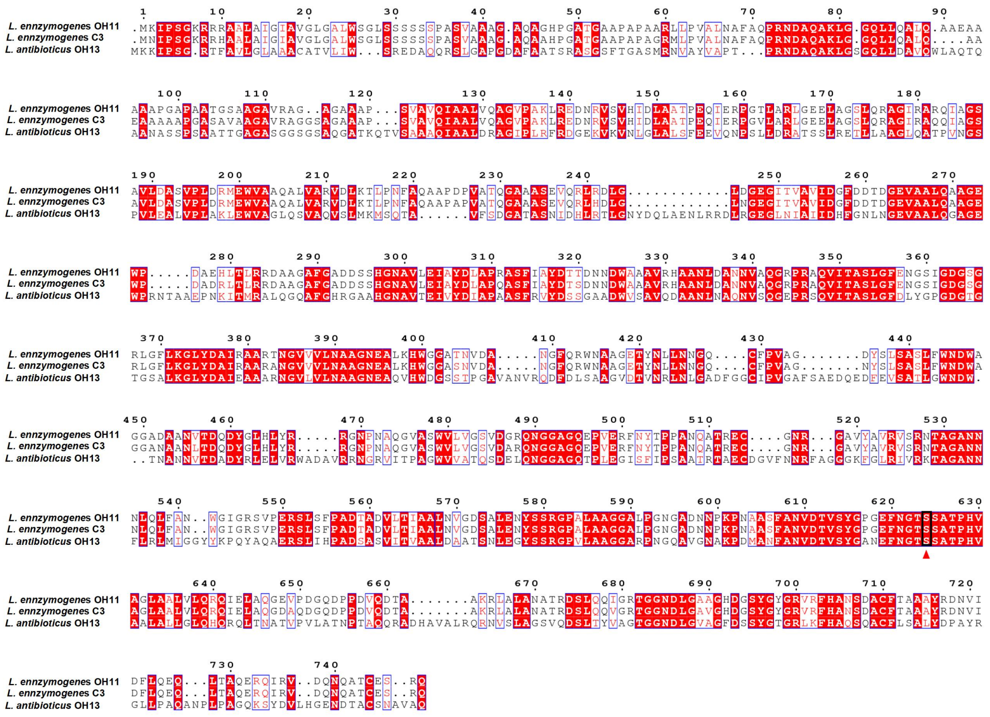

Figure S16. Sequence alignment of Le0100 and its two homologues (GLE3109 and La3673). The box indicates the position of the conserved amino acid residue S624. GLE3109 and La3673 are Le0100 homologs from *L. enzymogenes* C3 and *L. antibioticus* OH13, respectively.

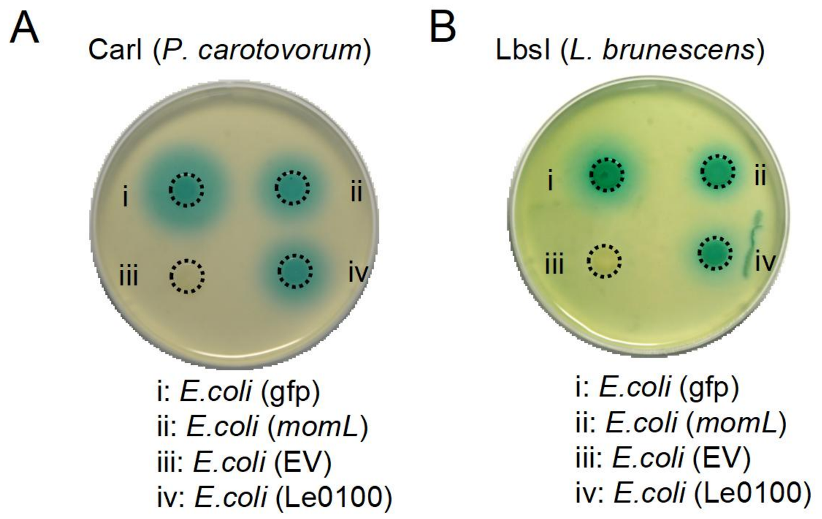

Figure S17. Representative images showing production of AHL in *E. coli* co-expressing Le0100 with the AHL synthase CarI (A) or LbsI (B) gene. AHL production was determined by the AHL biosensor JZA1. AHL quantification data in panels A and B were shown in Figure 4E and 4F, respectively.

References

1. Qian, G., Hu, B., Jiang, Y. & Liu, F. Identification and characterization of *Lysobacter enzymogenes* as a biological control agent against some fungal pathogens. Agricultural sciences in China 8:68-75. https://doi.org/10.1016/S1671-2927(09)60010-9.

2. Wei, H., and L. Zhang. 2006. Quorum-sensing system influences root colonization and biological control ability in *Pseudomonas fluorescens* 2P24. Antonie van Leeuwenhoek 89:267-280. https://doi.org/10.1007/s10482-005-9028-8.

3. Zhu, J., Y. Chai, Z. Zhong, S. Li, and S. C. Winans. 2003. Agrobacterium bioassay strain for ultrasensitive detection of N-acyl homoserine lactone-type quorum-sensing Molecules: Detection of Autoinducers in *Mesorhizobium* huakuii. Applied and Environmental Microbiology 69:6949-6953. https://doi.org/10.1128/AEM.69.11.6949-6953.2003.

4. Xu, G., S. Han, C. Huo, K. Chin, S. Chou, M. Gomelsky, G. Qian, and F. Liu. 2018. Signaling specificity in the c-di-GMP-dependent network regulating antibiotic synthesis in *Lysobacter*. Nucleic Acids Research. https://doi.org/10.1093/nar/gky803.

5. Kovach, M. E., P. H. Elzer, D. Steven Hill, G. T. Robertson, M. A. Farris, R. M. Roop, and K. M. Peterson. 1995. Four new derivatives of the broad-host-range cloning vector pBBR1MCS, carrying different antibiotic-resistance cassettes. Gene 166:175-176. https://doi.org/10.1016/0378-1119(95)00584-1.

6. Liao, J., D. Shen, L. Lin, H. Chen, Y. Jin, S. Chou, X. Yu, T. Li, and G. Qian. 2021. Bacterial quorum sensing quenching activity of *Lysobacter* leucyl aminopeptidase acts by interacting with autoinducer synthase. Computational and Structural Biotechnology Journal 19:6179-6190. https://doi.org/10.1016/j.csbj.2021.11.017.
